# tgLang: A Domain-Specific Language for Geometry Processing and Computational Imaging Workflows

**DOI:** 10.3390/jimaging12090406

**Published:** 2026-08-27

**Authors:** Vijai Kumar Suriyababu, Cornelis Vuik, Matthias Möller

**Affiliations:** Delft Institute of Applied Mathematics, Delft University of Technology, 2628 CD Delft, The Netherlands; c.vuik@tudelft.nl (C.V.); m.moller@tudelft.nl (M.M.)

**Keywords:** domain-specific language, computational geometry, geometry processing, mesh processing, image processing, computational imaging

## Abstract

Geometry-processing and computational-imaging workflows combine heterogeneous data structures, topology-changing edits, dense numerical fields, visualization, and repeated experimental variation. These workflows are often clear as algorithms but obscured in software by traversal boilerplate, representation conversions, build-system boundaries, and ad hoc scripting conventions. This paper presents *tgLang*, a domain-specific language with explicit, runtime-enforced representation types that makes meshes, point clouds, curve networks, grids, two-dimensional images, and image stacks first-class executable values. The language combines manifest types, typed arrays, modules, deterministic parallel constructs, flow-oriented queries, and runtime-provided domain operations. Its current implementation uses a stack-based bytecode virtual machine for reference semantics and dispatches representation-heavy operations to optimized C++ kernels. The evaluation is organized around complete workflows: topological hole detection, distance-field-based mean camber line extraction, voxel downsampling of point clouds, curve-network generation, surface-mesh smoothing and remeshing, image-stack edge detection, morphological image processing, and image-stack surface extraction. These examples show that a domain-aware source language can express multi-representation geometry and imaging algorithms as compact, reproducible programs while preserving explicit representation choices and a path toward deployable implementations.

## 1. Introduction

Preparing geometric data is still one of the most persistent practical costs in computational engineering workflows. Industrial geometries commonly arrive through Computer-Aided Design (CAD) systems with gaps, small features, non-manifold regions, or modeling details that are irrelevant to the simulation but problematic for meshing. The survey reported in [1] reflects this imbalance: a substantial fraction of engineering effort is spent before the analysis itself can begin. Robust meshing methods help, but their use in day-to-day pipelines often still requires case-specific scripting, repair steps, and manual parameter tuning.

Established C++ libraries, including CGAL [2], OpenMesh [3], OpenVolumeMesh [4], geometry-central [5], and libigl [6], provide high-quality data structures and algorithmic kernels. They are indispensable when robustness and performance are required. Their interfaces, however, place early-stage algorithm design inside low-level engineering concerns: template-heavy types, ownership conventions, traversal idioms, and conversion code can dominate a prototype before the core idea is visible. New methods also tend to cross library boundaries, which introduces build-system complexity and encourages project-specific wrapper layers that may be difficult to maintain.

High-level scripting environments address part of this problem. Python-based workflows, for example with PyMesh [7] or Open3D [8], are productive for exploration and data inspection. The tradeoff is that performance-sensitive kernels, stricter type contracts, and distributable implementations usually have to be introduced later through native extensions, separate executables, or a second implementation of the same algorithm.

### 1.1. Why a Language Rather than Another Library?

The motivation for a language layer is that research workflows repeatedly combine operations with very different programming styles: mutable surface-mesh edits, point-cloud filtering, grid-based scalar fields, curve-network construction, image and image-stack processing, file I/O, plotting, and repeated interactive testing. These concerns are algorithmically connected, but library-level implementations tend to expose them through separate APIs, traversal conventions, and conversion steps. The source program then records the mechanics of assembly as much as the algorithmic idea.

A dedicated source language gives these recurring elements a common representation. In *tgLang*, geometric values, image values, module boundaries, typed function signatures, deterministic parallel constructs, and runtime dispatch are visible in one program. The compiler and runtime can therefore attach diagnostics, type checks, scheduling rules, and domain-specific execution behavior to language constructs instead of relying on informal conventions distributed across scripts and wrappers.

The design follows a separation between the language and its execution machinery. *tgLang* is not a geometry library with a new calling syntax: its grammar, type system, module rules, flow semantics, diagnostics, and domain representations define a source language. tgVM is the reference backend of the present implementation, not the definition of the language itself. Programs currently compile to TGBC bytecode and delegate expensive domain operations to optimized C++ runtime kernels. The same grammar and source programs can instead target a bytecode-to-C++ backend or, for suitable data-parallel regions, a CPU/GPU backend, provided that the backend preserves the specified language semantics. These alternative backends are architectural directions rather than evaluated implementations in this paper. Table 1 contrasts this language-level model with conventional library-oriented workflow assembly.

### 1.2. Related Work

Established C++ libraries such as CGAL [2], OpenMesh [3], OpenVolumeMesh [4], geometry-central [5], and libigl [6] provide the kernels on which most practical geometry processing rests. They expose robust data structures and optimized algorithms, but their interfaces are optimized for performance and reliability rather than for rapid experimentation. Templates, ownership conventions, traversal idioms, and conversion code can dominate a small prototype.

High-level scripting environments partially address this friction. PyMesh [7] and Open3D [8] make inspection and small experiments productive, but performance-sensitive kernels, strict type contracts, and distributable implementations usually require a later rewrite into native extensions or separate executables. Gmsh [9] offers a mature scripting interface, yet it is tightly coupled to its meshing kernel rather than a general language for arbitrary mesh-processing algorithms.

Several DSL projects have attacked adjacent problems from different angles. Liszt [10] showed that finite-element computations on meshes can be expressed at a high level and compiled efficiently. Ebb [11] generalized this direction through a relational model separating simulation kernels, geometric-domain libraries, and CPU/GPU runtimes, while retaining static topology during a kernel phase. Simit [12] combines hypergraphs and tensor algebra for physical simulation and interoperates with C++ when topology or operations fall outside its graph abstraction. MeshTaichi [13] targets high-performance mesh operations through compile-time relation analysis and data-layout optimization, particularly for simulation and PDE-oriented workloads. I♥MESH [14] explores symbolic mesh-processing expressions and translation to several backends, including Eigen-based C++.

Imaging and sparse-computation DSLs address different optimization boundaries. Halide [15] separates the functional definition of an image pipeline from its schedule, enabling aggressive locality, vectorization, and heterogeneous execution without making mutable mesh topology a first-class concern. Taichi [16] decouples sparse spatial data-structure layout from imperative kernels and compiles for CPUs and GPUs; its principal abstraction is high-performance sparse fields and kernels rather than an end-to-end geometry-repair workflow. These systems are stronger than tgLang in automatic kernel compilation and accelerator targeting. tgLang instead targets orchestration across mutable meshes, point clouds, grids, curves, images, image stacks, plotting, and file I/O, with expensive operations delegated to runtime libraries.

Table 2 makes the distinction one of scope and execution rather than syntax. *tgLang* does not claim the schedule optimization of Halide, accelerator compilation of Taichi or MeshTaichi, or simulation-specific algebra of Simit. Its contribution is a single executable workflow layer spanning mutable geometry and computational imaging, including data loading, representation conversion, topology-changing algorithms, diagnostics, and visualization. The tradeoff is that generic bytecode-level scalar flows do not automatically attain the performance of those optimizing compilers; Section 4.7 reports the observed cost of complete *tgLang* workflows without making a general performance claim against those systems.

The comparison must also distinguish native DSL expressibility from what can be completed by escaping to a general-purpose host. Liszt explicitly fixes mesh topology during execution [10]; Ebb reports a static-topology implementation and invokes external libraries for operations outside its bulk-synchronous relational kernels [11]; and Simit states that topology changes are not expressible in the language, although its C++ Set API can modify a graph between executions [12]. I♥MESH likewise places procedural optimization algorithms and mutations such as edge flips outside its language scope and generates routines for invocation by a host program [14]. Consequently, the longest-edge subdivision and isotropic-remeshing workflows in this paper cannot be expressed wholly inside any of these reported DSL models. MeshTaichi provides efficient kernels and relation queries over mesh elements [13], but its reported programming model does not provide the dynamic split–collapse–flip connectivity updates required by those workflows.

Other examples cross representation boundaries rather than only mutating topology. Mean-camber extraction moves from an airfoil curve to a signed-distance grid, field derivatives, grouped samples, and a reconstructed curve; implicit-surface extraction moves from a three-dimensional image stack to a boundary triangle mesh and then mesh smoothing; hole detection ends a mesh-connectivity analysis with component-to-curve conversion and visualization; voxel downsampling performs dynamically keyed regrouping followed by reconstruction of a point-cloud value; and the polar-web example constructs, filters, combines, and plots collections of curve networks. Halide natively addresses feed-forward image and tensor pipelines [15], while Taichi addresses kernels over dense or sparse fields and particles [16]; neither reported model provides these complete cross-representation geometry workflows without host-side data structures and calls. Conversely, fixed-topology smoothing is directly aligned with Liszt, Ebb, Simit, MeshTaichi, and I♥MESH, and the stack-edge and morphological-closing kernels fit Halide or Taichi. Thus, the distinction is not that the underlying mathematics is impossible elsewhere, but that at least seven of the ten evaluated workflows require a key stage outside every comparator’s native DSL abstraction.

**Figure 1:**
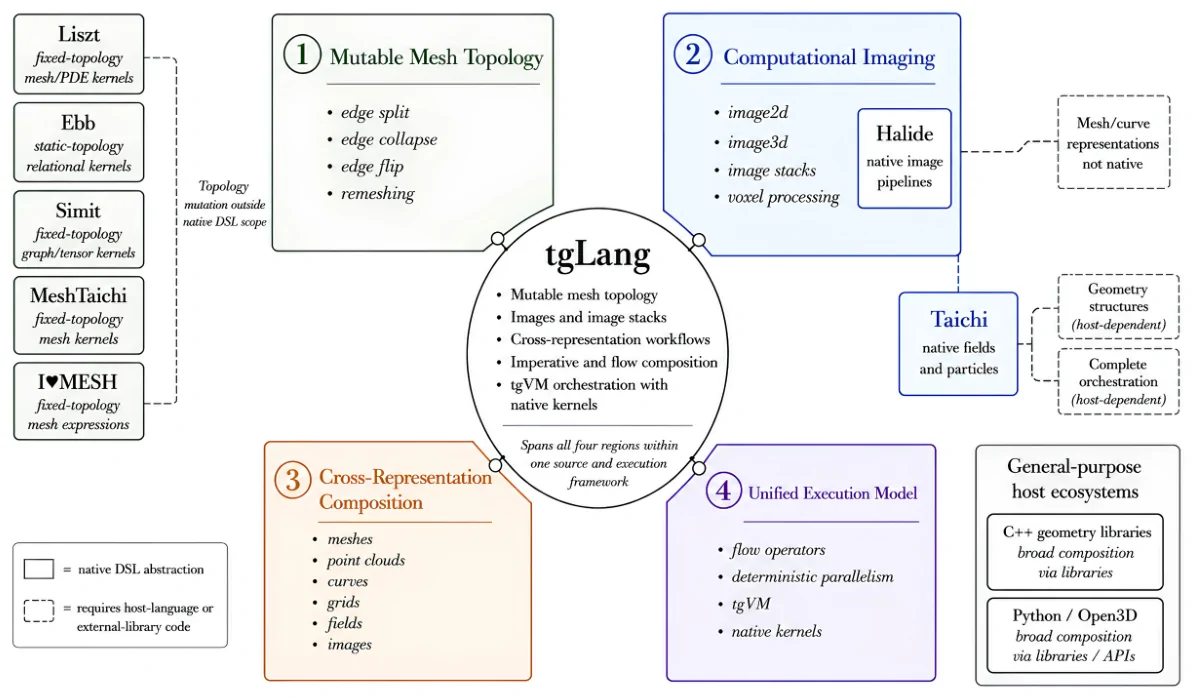
Capability landscape of the compared systems. Solid boundaries denote facilities native to the stated programming model, whereas dashed boundaries denote stages requiring host-language or external-library code. The diagram summarizes scope rather than performance; Table 2 and the cited primary publications specify the corresponding system boundaries.

Figure 1 summarizes these native abstraction boundaries and the four capabilities exercised jointly by the evaluated workflow set.

The main contributions are as follows:

- **Geometry- and image-native manifest typing:** *tgLang* includes dedicated types for pointCloud, cubeGrid, surfaceMesh, curveNetwork, image2d, image3d, volumeMesh, and related objects, in addition to primitive and array types. These declarations make representation assumptions visible in function signatures and intermediate values, while tgVM validates dynamically selected native operations and data-dependent invariants.
- **A module model for domain operations:** Functionality is organized through modules and can be extended with extendModule, allowing experimental routines to be packaged under the same naming and type-checking rules as built-in operations.
- **Flow-oriented algorithm composition:** Recent versions of the compiler add pipeline expressions, context binding, side-effect taps, grouping, filtering, concatenation, and module-qualified pipeline calls. These constructs make complete geometry and imaging workflows read as transformations of domain values rather than as sequences of temporary containers.
- **Reproducible parallel semantics:** Supported parallel constructs use deterministic partitioning and collection rules, which makes repeated runs easier to compare during debugging, validation, and figure generation.
- **Runtime-kernel integration:** The implementation combines a bytecode virtual machine with C++ runtime kernels for domain operations, providing a path from interactive scripts to reproducible batch workflows without requiring algorithms to be rewritten in a host language.

The remainder of the paper is structured as follows. Section 2 defines the language model and the geometry- and image-oriented type system. Section 3 describes execution through the virtual machine and runtime services. Section 4 presents representative workflows, and Section 5 and Section 6 discuss design implications and summarize the work.

## 2. Language Design

The design of *tgLang* starts from the fact that many geometry and imaging programs are not just numerical loops over arrays. They maintain connectivity, inspect neighborhoods, update topology, transfer data between representations, and combine meshes, point clouds, curve networks, Cartesian grids, images, and image stacks in one workflow. General-purpose languages can express all of these steps, but the domain meaning is usually encoded indirectly through library-specific classes and traversal conventions. *tgLang* instead exposes the common representations directly in the language type system.

Most of the surface language is intentionally conservative. Variables carry declared or inferred representation types, functions declare argument and return types, control flow follows a C-like style, and modules provide namespaces. Validation is deliberately hybrid: the compiler records declarations, resolves source names, and enforces structural restrictions, whereas tgVM selects native overloads and validates representation and shape constraints against the values that reach a call. The domain-specific aspect is therefore not unusual syntax; it is the combination of familiar imperative programming with first-class geometry and image values, typed arrays, module extension, flow-oriented queries, and deterministic execution rules. Table 3 summarizes the representative built-in types.

Array types are written in the source language rather than inherited from C++ templates. For instance, array<double,{3}> denotes a three-component double array, while higher-rank forms support grid fields, tensors, and dense numerical data. When the element type or shape is inferable, a shorter array spelling can be used; internally, however, the compiler still works with a concrete typed array.

### 2.1. Type Guarantees and Dynamic Validation

The terms *explicitly typed* and *strongly typed* require a precise boundary in the current implementation. A source declaration fixes the intended representation and that annotation is retained during bytecode generation; values do not silently reinterpret a surfaceMesh as a pointCloud, a string as a number, or an image as an array. The present compiler is not, however, a whole-program static type prover. Native functions and methods are overload-resolved from the runtime types of their arguments, and constructors validate the received values when the corresponding bytecode executes. Consequently, an invalid native call can pass source compilation and then raise a source-mapped tgTypeError or overload error before the operation is performed. We use *manifest typing with hybrid validation* for this model and do not claim that all type errors are rejected by –check. Table 4 states the compile-time and runtime boundary explicitly.

For example, the native generateSphere overload registered for arguments (int, double, array) rejects a string-valued first argument during runtime overload selection. Likewise, pointCloud construction accepts only a numerical N×2 or N×3 array (or a compatible collection) and reports other ranks or widths dynamically. Mesh manifoldness, valid edge identifiers, property lengths, and image-stack dimensions depend on loaded data and are runtime invariants by design. This separation makes the guarantees explicit without presenting data-dependent geometry validity as a static property. Listing 1 illustrates the corresponding explicit function signature and array shape.

**Listing 1:** A minimal typed function in *tgLang*.

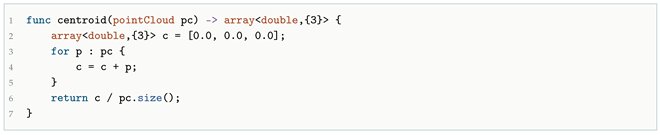



Modules collect related operations and provide a route from local experimentation to reusable functionality. Geometry-processing code often starts as a small helper routine attached to one dataset or figure; if it becomes stable, extendModule allows it to be placed under an existing namespace while keeping an explicit signature.

The resulting source remains compact, but the receiver type, return type, and namespace are still recorded. The same pattern is used for point-cloud routines, grid computations, curve-network algorithms, image filters, image-stack projections, and mutation operations on meshes.

The language is designed for gradual structure. A compact prototype can use built-in domain types, loops, arrays, and existing modules; as the method stabilizes, module extensions and user-defined record-like values provide names, signatures, and reusable packaging for the emerging operation. The same source language therefore covers both one-off exploratory scripts and reusable workflow components.

### 2.2. Core Syntax

The surface syntax is intentionally conservative: explicit typed declarations, C-like control flow, functions with argument and return types, modules with aliases, and structured error handling. This is a design decision. By keeping the imperative shell familiar, the language can direct attention to the domain-specific parts of a program: first-class geometric and image types, typed arrays, module extension, and deterministic parallel and flow constructs. Listing 2 shows how an experimental routine is attached to an existing module; small structured values are defined with special for lightweight record-like algorithmic settings; and try/catch exposes data-dependent failures such as non-manifold input or empty geometry at the source level rather than through unchecked status values. The workflow examples in Section 4 therefore read as executable algorithms rather than as thin wrappers around low-level API calls.

**Listing 2:** Extending the surface-mesh module with a derived quantity.

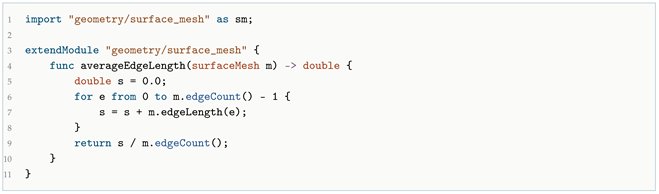



### 2.3. Flow-Oriented Composition

The compiler includes a flow-oriented layer on top of the typed language. The purpose is to remove the temporary-variable scaffolding that often obscures geometry and image-processing algorithms while retaining ordinary functions, modules, and explicit types. A flow expression treats the value on the left of a dot as the current receiver and passes it through a sequence of transformations, filters, grouping operations, module functions, and side-effect taps. The resulting notation is still compiled by the same front end and executed by tgVM; the compiler lowers the additional syntax to ordinary bytecode calls, temporary locals, and checked runtime dispatch.

This layer is useful when an algorithm is naturally described as a refinement of a domain set. For example, a surface mesh can produce an edge stream, the stream can be filtered by feature angle and curvature, connected components can be formed, and the surviving components can be converted to curve networks for plotting. The same form applies to scalar-field samples, point-cloud samples, image pixels, and image-stack voxels: the program describes what is selected, grouped, refined, converted, and visualized, while the runtime provides the representation-specific kernels. Table 5 summarizes these operators and their compiler-level behavior.

The implementation deliberately keeps these operators generic. The grammar does not expose separate public types such as edgeSelection, fieldSample, pixelSelection, or voxelSelection; such intermediate values are internal runtime values produced by methods and consumed by flow operators. This preserves explicit representation types for user-visible values while giving the compiler and runtime enough context to interpret expressions such as mesh.edges().where(e => ...), field.groupBy(s => s.index.x), or image.pixels().where(p => ...) in a domain-aware way.

The compiler changes are small but important. Expression-level with, tap, apply, repeat, and module-qualified pipeline calls are parsed directly. Element-wise flow operators such as where, all, map, groupBy, maxBy, and each are lowered to ordinary bytecode loops over runtime-provided iterators. During lowering, the compiler binds the lambda parameter to the current element and records any internal entity context, such as selected mesh edges, selected vertices, connected edge components, scalar-field samples, image pixels, or image-stack voxels. tap is lowered specially so that it evaluates its lambda for side effects and then reloads the original receiver. Module lookup also supports an any-typed fallback for flow utilities such as console taps and plot sinks, which avoids duplicating overloads for every geometry type. These choices keep the surface syntax concise while preserving the existing VM execution model.

## 3. Implementation and Execution Model

The current implementation is centered on the *tgLang* virtual machine (tgVM), which executes stack-oriented bytecode and defines the observable runtime behavior. Source files and imports are first resolved into a checked program representation. Lexing and parsing use ANTLR4 [17,18]; subsequent semantic analysis emits bytecode that is executed by tgVM and connected to runtime modules, type methods, and optimized C++ domain kernels.

**Figure 2:**
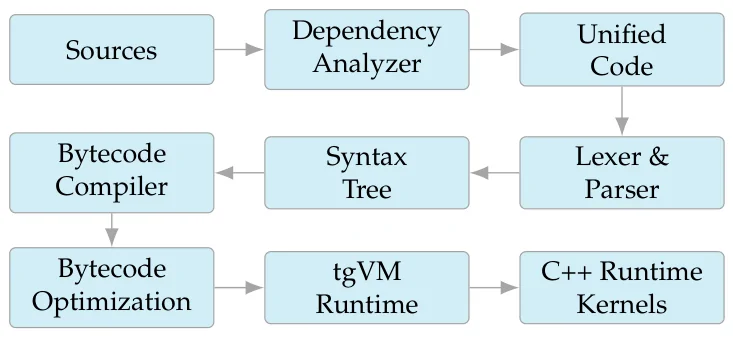
Current compilation and execution pipeline used to resolve *tgLang* sources, generate bytecode, and dispatch domain operations to C++ runtime kernels.

The bytecode path keeps the intermediate representation compact. Dependency resolution produces a unified source view, parsing builds the syntax tree, semantic analysis constructs the checked program, and the compiler emits *tgLang* bytecode (TGBC). The emitted module records local variables, constants, imports, module variables, and source-map information; simple optimization passes can fold constant expressions, remove unreachable instructions, and simplify redundant method-call patterns before execution. Figure 2 summarizes this compilation and execution path.

### 3.1. Bytecode Virtual Machine

tgVM provides the reference implementation of the language semantics. A program is compiled to stack-based TGBC, serialized as a module, and loaded by a runtime that stores functions, modules, type methods, source maps, and native-kernel bindings. Execution is organized around an operand stack, local-variable storage, a constant pool, and explicit instruction pointers. Source maps connect bytecode locations back to the original program, which is important when diagnosing failures in exploratory scripts. Neither the grammar nor the source-level workflow model requires interpretation: TGBC can also serve as a typed lowering boundary for native C++ generation, and data-parallel operations can be lowered to accelerator kernels without changing the source program. Those routes must preserve tgLang’s observable type, error, ordering, and mutation semantics.

The VM is used as the reference route for engineering rather than because stack interpretation is expected to outperform compiled numerical code. A single bytecode format gives batch, server, REPL, and WebAssembly deployments the same module and error semantics; source maps remain available after dependency unification; and flow operators can be lowered without requiring a second source language. The cost is dispatch and allocation overhead for fine-grained bytecode operations. Section 4.7 therefore reports complete-workflow timings across the evaluated workload set. Coarse representation-heavy operations remain native, while long chains of scalar map or groupBy work expose the current VM overhead directly.

tgVM defines language semantics rather than reimplementing every geometry or imaging kernel at the bytecode level. Calls that require substantial domain work, such as mesh connectivity queries, point-cloud neighborhood searches, image morphology, image-stack projection, array kernels, or grid stencils, are dispatched to optimized C++ implementations. This keeps the source program compact without forcing mature geometry and imaging kernels to be rewritten inside the interpreter.

The instruction set is intentionally small enough to inspect. Module files can be disassembled, and individual instructions carry source-location information for error reporting. A representative subset of TGBC instructions is shown in Table 6.

Flow syntax does not introduce a separate bytecode language. For example, where lowers to a loop that stores the receiver in a temporary local, calls __vm_iterLength and __vm_iterGet, evaluates the predicate body with the lambda parameter bound to the current element, and appends surviving elements to a representation-compatible output. Similarly, groupBy uses __vm_flowEmptyGroups and __vm_flowGroupAdd, while tap clones the receiver for observation and reloads the original value for the next stage. This design keeps the bytecode VM stable while allowing the compiler and runtime to add higher-level flow behavior.

### 3.2. Garbage Collection

tgVM can manage language-level objects with a precise generational garbage collector. Each runtime owns its heap, which avoids sharing allocator state across workers. The collector enumerates roots from the operand stack, local variables, bytecode constants, module variables, and special initializers. A write barrier records old-to-young references so that minor collections can be performed efficiently.

Meshes, point clouds, arrays, and tables appear as managed language values, but their large payloads remain inside type-specific runtime structures. The collector therefore controls object lifetime for script-visible values, temporary records, closures, tables, and references without treating every geometry buffer as a scalar object. For parallel execution, worker heaps remain isolated; returned values are adopted into the parent heap by traversing the reachable object graph rather than by deep-copying every object.

Garbage collection is disabled by default and enabled explicitly with the –garbageCollect command-line flag. In the default mode, allocated language objects remain registered with their runtime heap and are reclaimed together when that runtime is destroyed; unreachable temporaries are not reclaimed during execution. This is appropriate for short batch programs but can increase peak memory in a long-lived REPL, server session, or allocation-heavy workflow. With collection enabled, a minor cycle is considered after 20,000 allocations, a major cycle after 100,000 allocations, and objects surviving two minor cycles are promoted. The measured time and peak-resident-memory effects for point-cloud and volumetric workloads are reported in Section 4.7. Native mesh, image, point-cloud, and array payloads retain their type-specific ownership and allocation strategies in either mode.

### 3.3. Deterministic Parallel Execution

**Figure 3:**
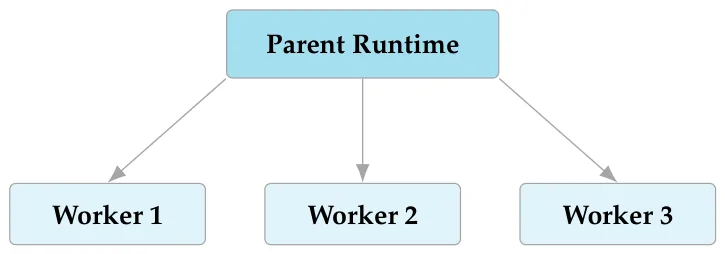
Deterministic parallel execution using cloned worker runtimes and fixed-order result collection.

Determinism is guaranteed only for the supported data-parallel subset. Work is partitioned by stable input ranges, worker runtimes are cloned from the parent runtime, per-element results are placed at their input indices, and supported keyed reductions merge worker partials in worker-index order. Direct writes to captured non-local variables are rejected in parfor. Arbitrary shared mutation is not deterministic, and topology-changing mesh operations remain serial unless an algorithm supplies an explicit conflict-free partition. Floating-point results can still reflect the documented merge order and should not be interpreted as order-independent real arithmetic. This bounded contract is useful for geometry-processing experiments, where scheduling differences can otherwise obscure whether a change came from the algorithm or from the execution order. Figure 3 shows the worker-runtime structure.

When garbage collection is active, tgVM suspends collection during parallel regions. After the region finishes, worker results are adopted by the parent heap, which preserves reachable object structure without recursively cloning every returned value. Parallel startup and collection are nevertheless not free: each worker requires cloned runtime state and worker-local allocations, and the parent adopts reachable results and merges them in order. These costs explain why parallel speedup depends on work per element and output size rather than only on processor count. The evaluation verifies byte-for-byte identical serialized outputs for fixed-input map and keyed-reduction programs at 1, 2, 4, and 10 workers; randomly generated inputs are intentionally excluded from that test. This is an intra-platform scheduling guarantee, not a claim of byte-identical floating-point serialization across different processors or operating systems.

### 3.4. Runtime Kernels and Type Methods

tgVM is the language runtime, not the numerical geometry or image kernel. It owns source-level semantics, bytecode execution, dispatch, diagnostics, and object lifetime, while representation-heavy operations remain in runtime modules and type methods implemented in C++. A method such as mesh.edges(), edge.anhedralDeg(), image.close(), or stack.mipZ() is therefore visible and typed in the source program, but its numerical or topological work is performed by the appropriate runtime data structure.

This boundary is important for the flow layer. The compiler can lower where, map, groupBy, tap, and module-qualified pipeline calls uniformly, while the runtime preserves domain context for internal stream values. Mesh-edge streams retain access to their parent mesh, field samples retain grid coordinates and derivative values, image pixels retain their raster coordinates, and image-stack voxels retain slice indices. The source syntax remains generic, but the runtime has enough information to perform representation-specific operations correctly.

### 3.5. Extension Model

There are two extension levels. A source-level module or extendModule block, illustrated in Listing 2, is compiled with the importing program and requires no modification or rebuild of tgVM. It can combine existing values and operations, define overloads with explicit signatures, and be distributed as ordinary .tg source. A native extension adds a C++ module function or type method to the runtime registry. The registration stores a return type, ordered argument types, and a callback; those signatures participate in runtime overload selection and source-mapped diagnostics. Listing 3 shows the essential pattern without the surrounding header and module-installation boilerplate.

**Listing 3:** Minimal native module registration pattern. Native extensions are compiled into
the current runtime.

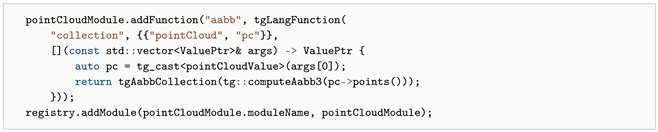



**Figure 4:**
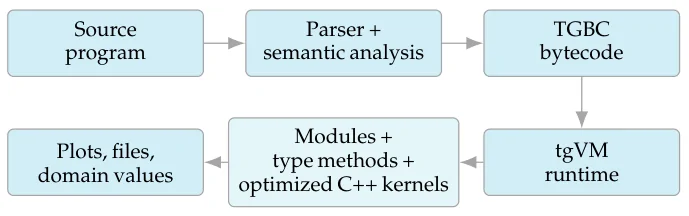
Current execution model: tgVM defines language semantics while runtime modules and type methods provide optimized geometry and imaging kernels.

Native registration currently requires rebuilding tgLang; the implementation does not claim a stable binary plugin ABI. Adding an algorithm over an existing representation requires its callback and registration signature. Adding a new native value representation additionally requires a value class, runtime type identifier, construction and conversion rules, tracing/cloning behavior for managed references, method registrations, and runtime recompilation. This distinction is important: tgLang source modules provide lightweight algorithm extensibility, whereas native representation extensibility remains a C++ integration task. The evaluation separates language-level orchestration from native-kernel work so that compact source is not presented as evidence that bytecode itself is faster than a specialized library. Figure 4 summarizes the resulting runtime-kernel boundary.

The validation set for the current implementation therefore focuses on VM execution of complete scripts. It includes scalar language features, arrays, modules, parallel constructs, mesh and point-cloud operations, curve networks, grids, image and image-stack operations, I/O, and visualization. The implementation claim is that tgVM and the runtime can execute full geometry and imaging workflows reproducibly today; additional native-code targets are an extension path, not a prerequisite for the results reported here.

### 3.6. Interactive and Browser-Based Execution

Besides batch execution, *tgLang* includes an interactive Read-Eval-Print Loop (REPL). The REPL accepts multi-line compound statements and prints expression values when they are not terminated by a semicolon. Introspection commands such as :help, :modules, and :types expose available modules, functions, and type methods from inside the session; shell commands can be invoked with the ! prefix.

**Figure 5:**
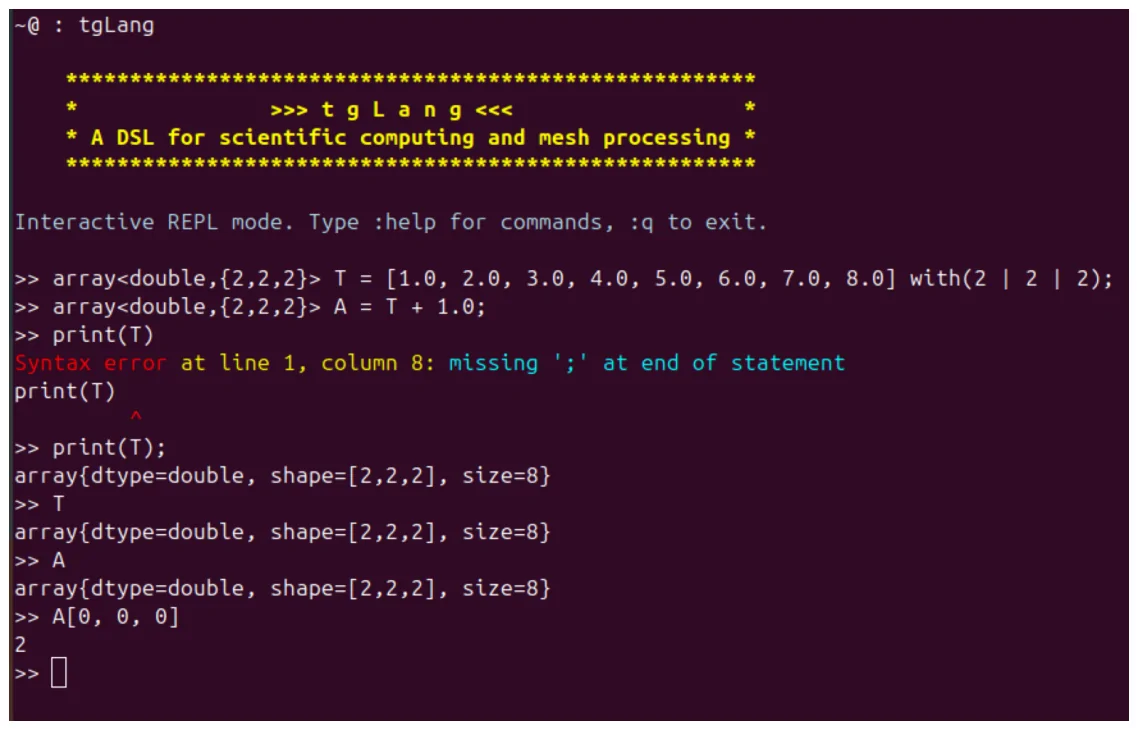
Interactive *tgLang* session with multi-line input and expression-value printing.

**Figure 6:**
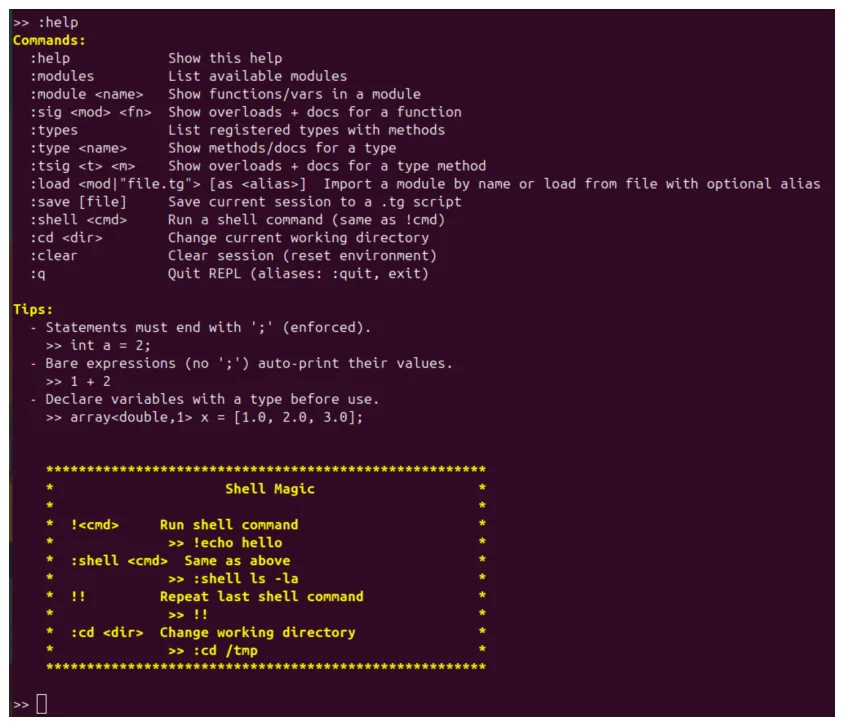
REPL introspection for module discovery and function-signature inspection.

The interactive mode supports the way many geometry workflows are developed: inspect available operations, test a small expression, load data, examine an intermediate object, and then move the stable sequence into a script. This keeps early exploration inside the same language used for the final workflow. Figure 5 and Figure 6 illustrate interactive evaluation and introspection.

**Figure 7:**
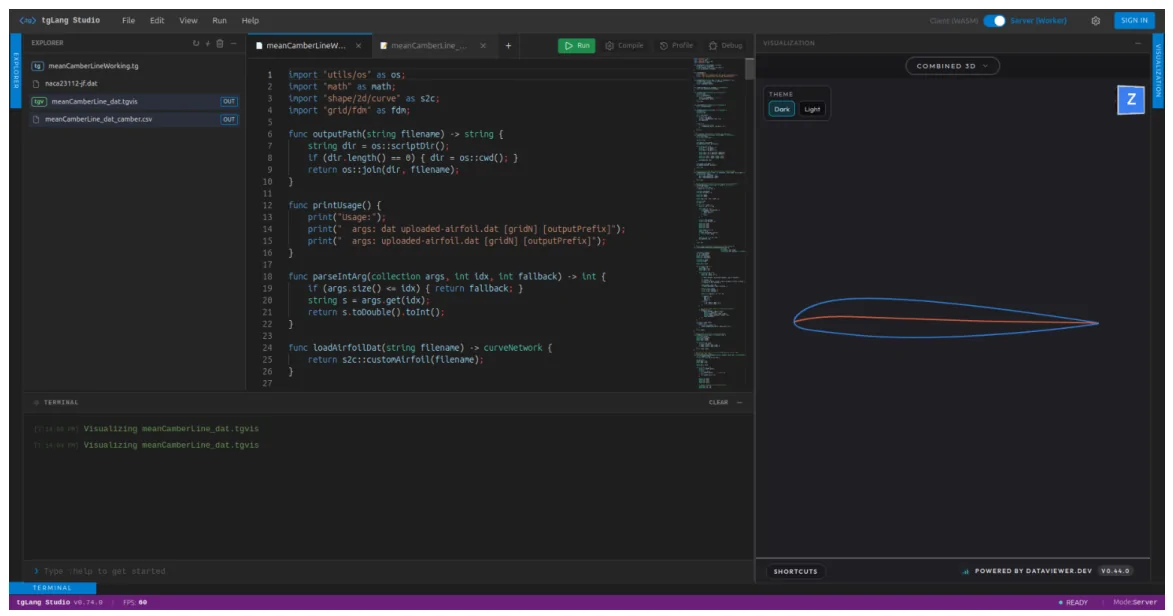
*tgLang Studio* browser interface with source code, generated files, terminal output, and integrated geometry visualization.

The same programming model is available in *tgLang Studio*, a browser-based interface with source editing, execution output, generated files, and geometry visualization. This lowers the barrier for sharing examples with collaborators, students, or reviewers, since the script can be inspected and run without reconstructing a local development setup. The browser interface is a front end to the same typed language rather than a separate execution model, as shown in Figure 7.

## 4. Representative Geometry and Imaging Workflows

The examples in this section are complete research workflows rather than isolated syntax fragments. They cover the representation and execution categories in Table 7: read-only topology queries, mutable topology, neighborhood geometry, generic grouping, dense scalar fields, raster and volumetric processing, native kernels, bytecode-heavy flows, serial mutation, and deterministic data parallelism. Controlled synthetic inputs make transformations visually unambiguous, while the hole-detection and remeshing cases include externally sourced meshes. For reproducibility, the workflow implementations and redistributable input data are distributed with the artifact, and the reported outputs can be regenerated in one batch.

Table 8 reports objective structural metrics for the complete executable source programs rather than the shortened manuscript listings. Source lines of code exclude blank and comment-only lines; function and import counts use declarations in the source; and flow stages count invocations of the compiler-supported composition operators listed in Table 5. These measures describe the visible program structure only. They are not used as proxies for development effort, readability, or programmer productivity.

### 4.1. Surface-Mesh Workflows

#### 4.1.1. Topological Hole Detection

Hole detection is a compact algorithmic idea that still requires substantial mesh infrastructure in a general-purpose library. The workflow depends on half-edge connectivity, feature-angle measurements, curvature-based filtering, loop extraction, and geometric postprocessing. The example follows the topological hole-detection workflow developed in prior work [19,20], which separates intended through-holes or tunnel-like features from accidental missing faces.

**Figure 8:**
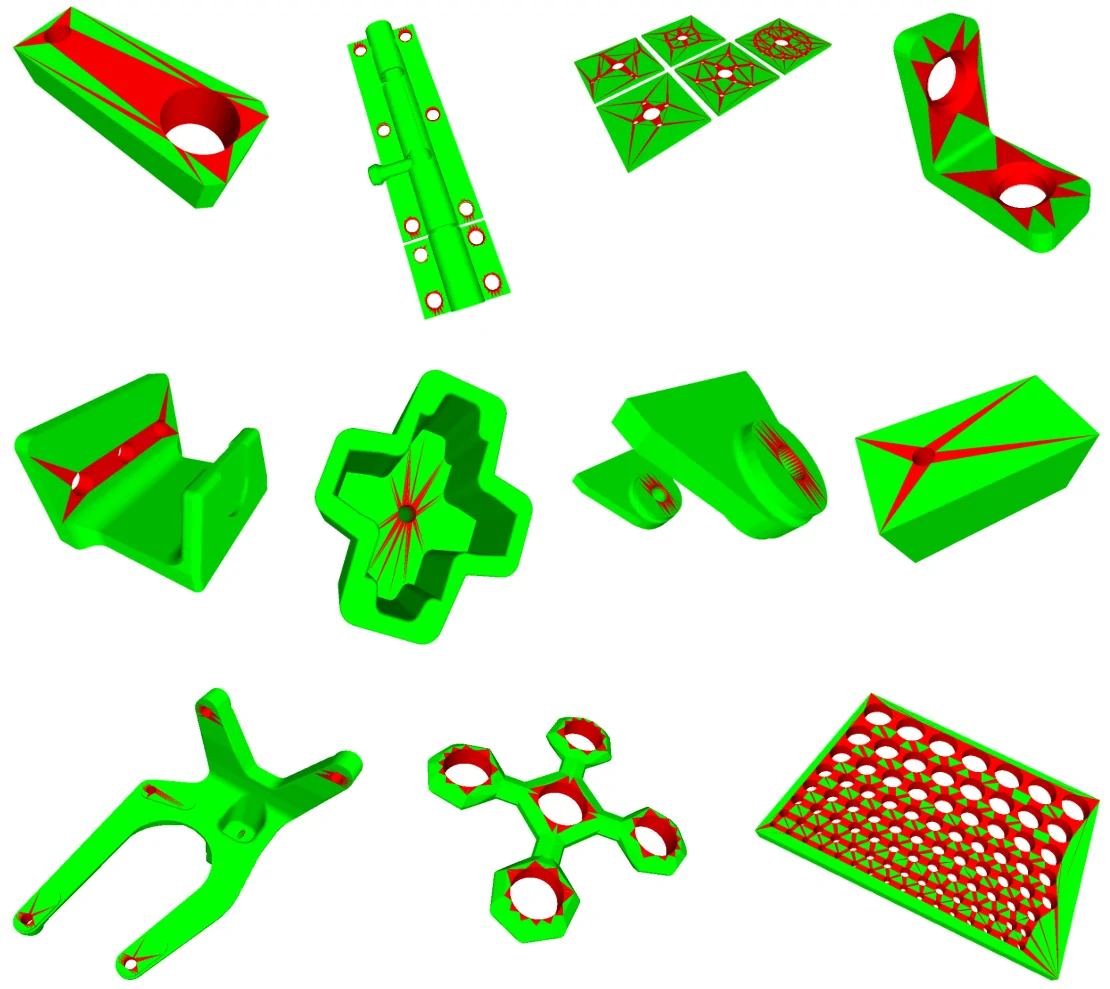
Detected hole boundaries on representative meshes, adapted from the thesis workflow in [20] and reproduced here as a *tgLang* script combining connectivity queries, feature-angle tests, curvature filtering, and loop extraction. The datasets used for these representative results are available through the associated Mendeley Data artifact [21].

Listing 4 shows the flow version of the complete script. It narrows the candidate set with anhedral-angle and integrated-curvature tests, enforces degree-two local connectivity, forms connected components, keeps only cycles, converts them to curve networks, and sends the result to the plotting module. The contribution of the language is the executable formulation: the script refers to mesh entities and domain operations directly, instead of expanding the method into iterator and connectivity boilerplate. Figure 8 shows representative detected boundaries.

The example also illustrates why complete workflows matter for evaluating the language. The underlying method and its engineering context are documented independently [19,20]; *tgLang* supplies a typed source representation through which such a method can be expressed, inspected, and rerun.

**Listing 4:** Flow-style *tgLang* script for feature-edge hole detection.

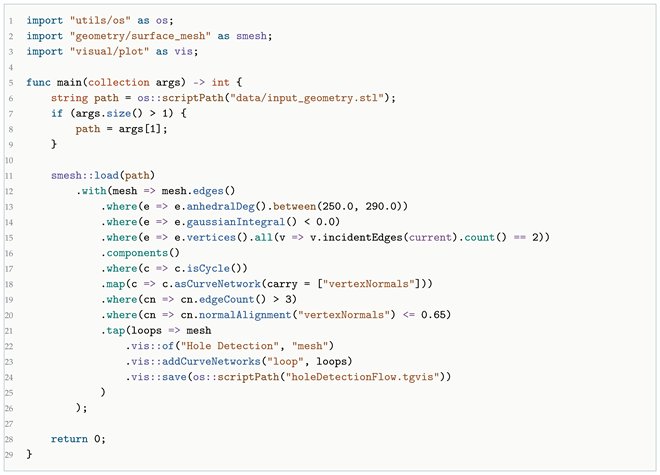



#### 4.1.2. Surface Smoothing

**Figure 9:**
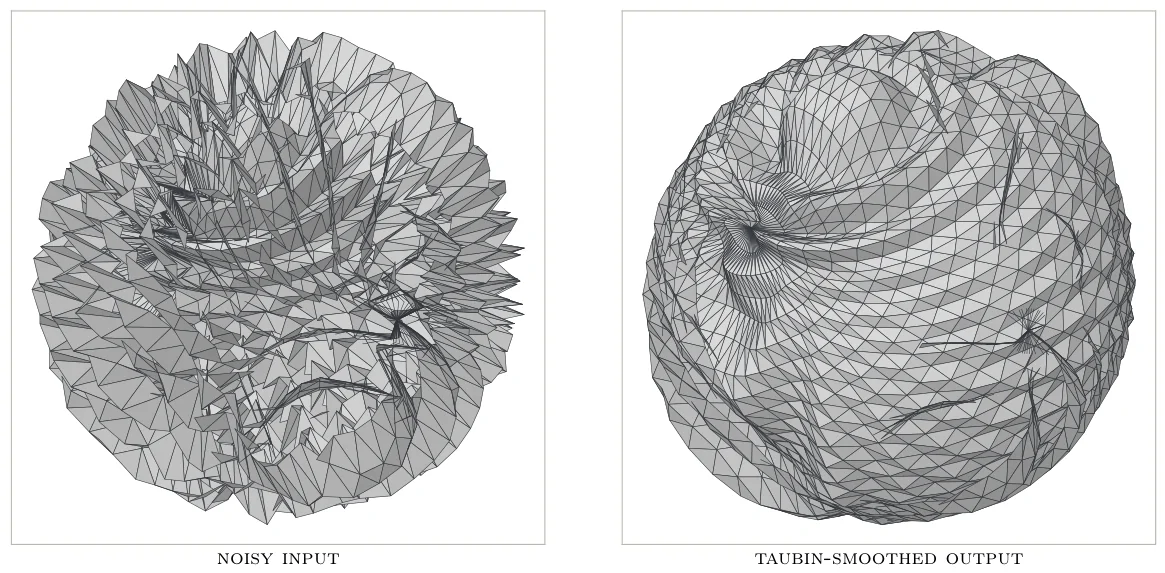
Surface-mesh smoothing expressed as a *tgLang* workflow over the native mesh type. A noisy synthetic sphere is smoothed by repeated Taubin-style positive and negative displacement passes.

For surface meshes, the surfaceMesh type exposes both mutation routines and differential operators. The algorithmic excerpt in Listing 5 evaluates a normalized neighborhood displacement and applies two passes per iteration: a positive pass and a compensating negative pass to limit shrinkage. Argument handling, validation, and visualization remain in the complete artifact source but are omitted from the manuscript excerpt. Figure 9 shows the resulting smoothing behavior.

**Listing 5:** Algorithmic core of Taubin smoothing with a normalized neighborhood operator.

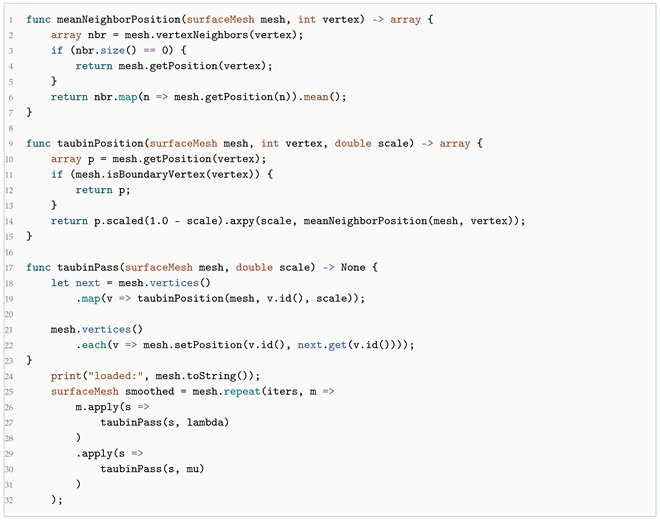



#### 4.1.3. Longest-Edge Subdivision

**Figure 10:**
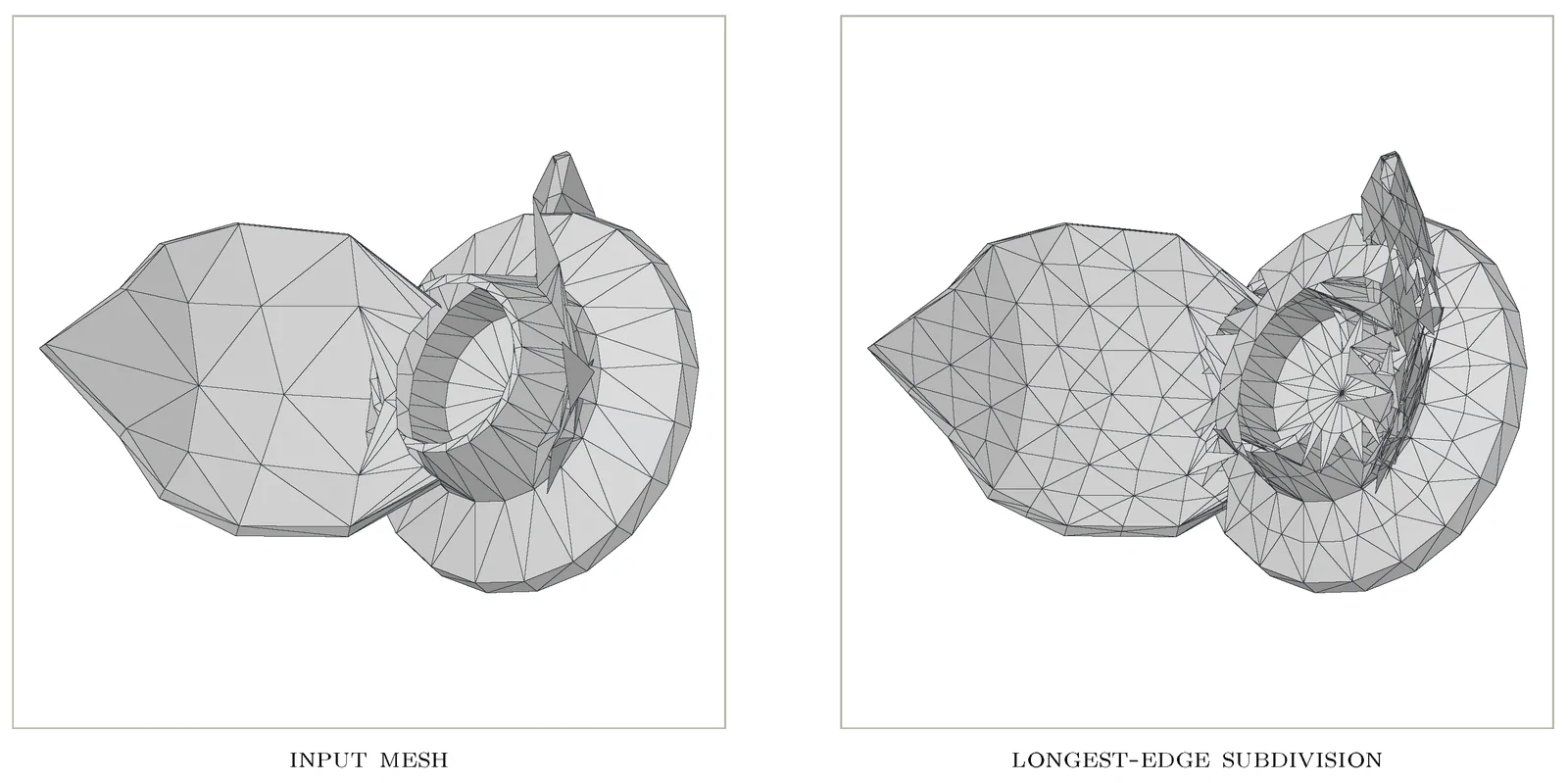
Longest-edge subdivision on a coarse surface mesh. The algorithm repeatedly splits the current longest edge above the target length, producing a denser triangulation while retaining the input geometry.

The same surface-mesh abstraction is also used for topology-changing workflows. Listing 6 implements longest-edge subdivision directly in the language. It derives a target length from the mean edge length when none is supplied, selects overlong edges through a flow query, maintains an explicit work queue, and splits the currently longest edge until the queue is empty. This exposes the algorithmic control flow rather than hiding refinement behind a single runtime call. Figure 10 shows the resulting refinement.

**Listing 6:** *tgLang* script for queue-driven longest-edge subdivision.

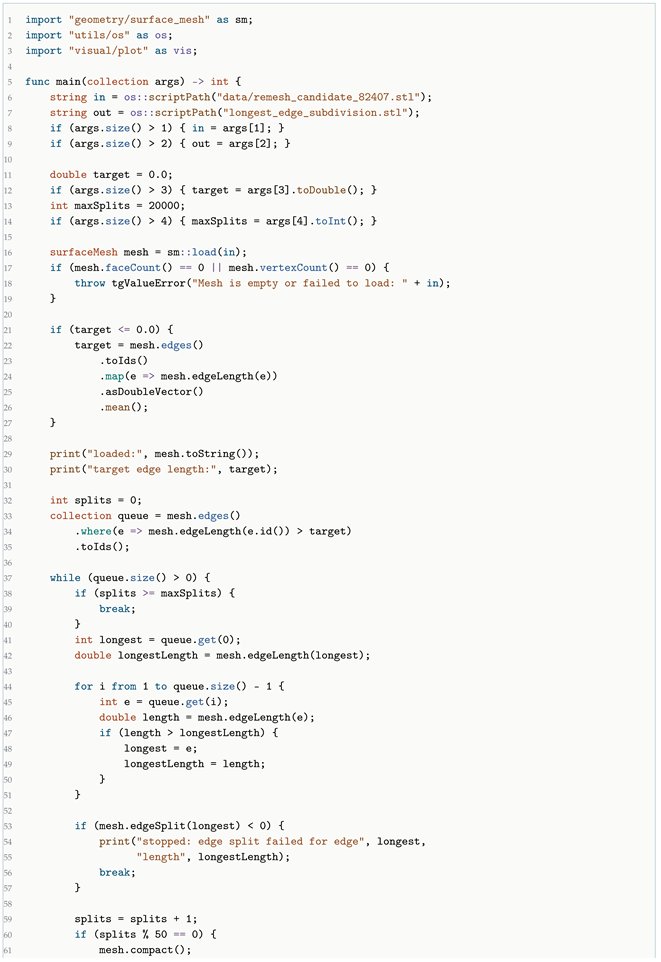



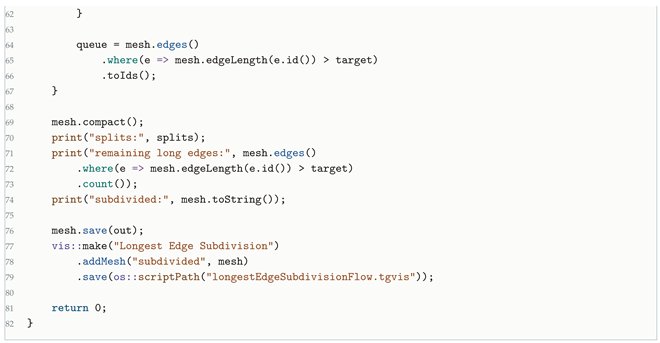



#### 4.1.4. Isotropic Remeshing

**Figure 11:**
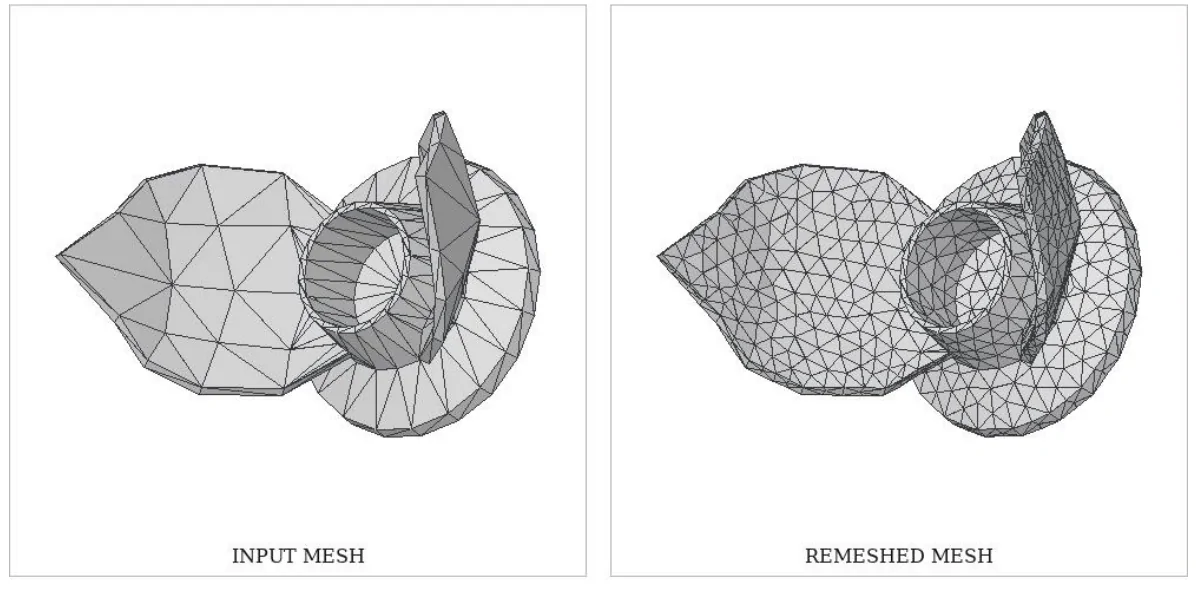
Isotropic remeshing workflow on a coarse demonstration mesh, showing conservative feature-preserving refinement produced by the *tgLang* script.

The isotropic remeshing workflow extends refinement with edge collapses and flips, tangential smoothing, and projection to the reference surface. Listing 7 retains the complete topology-changing pipeline while omitting command-line argument and output-handling boilerplate. Figure 11 compares the input and remeshed surfaces.

**Listing 7:** Algorithmic core of the *tgLang* isotropic surface-remeshing workflow.

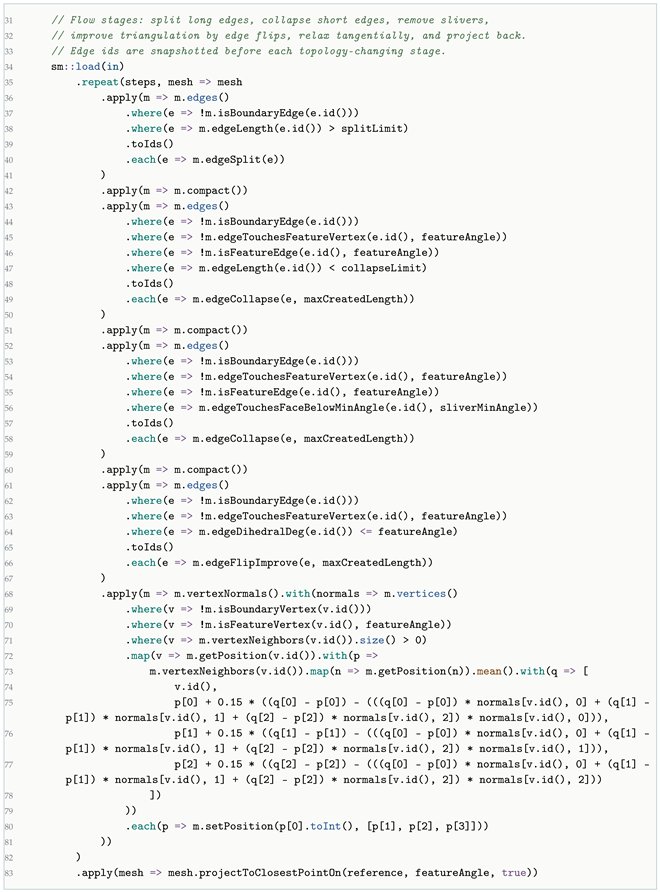



### 4.2. Grid and Scalar-Field Workflows

#### Distance-Field-Driven Mean Camber Line Extraction

**Figure 12:**
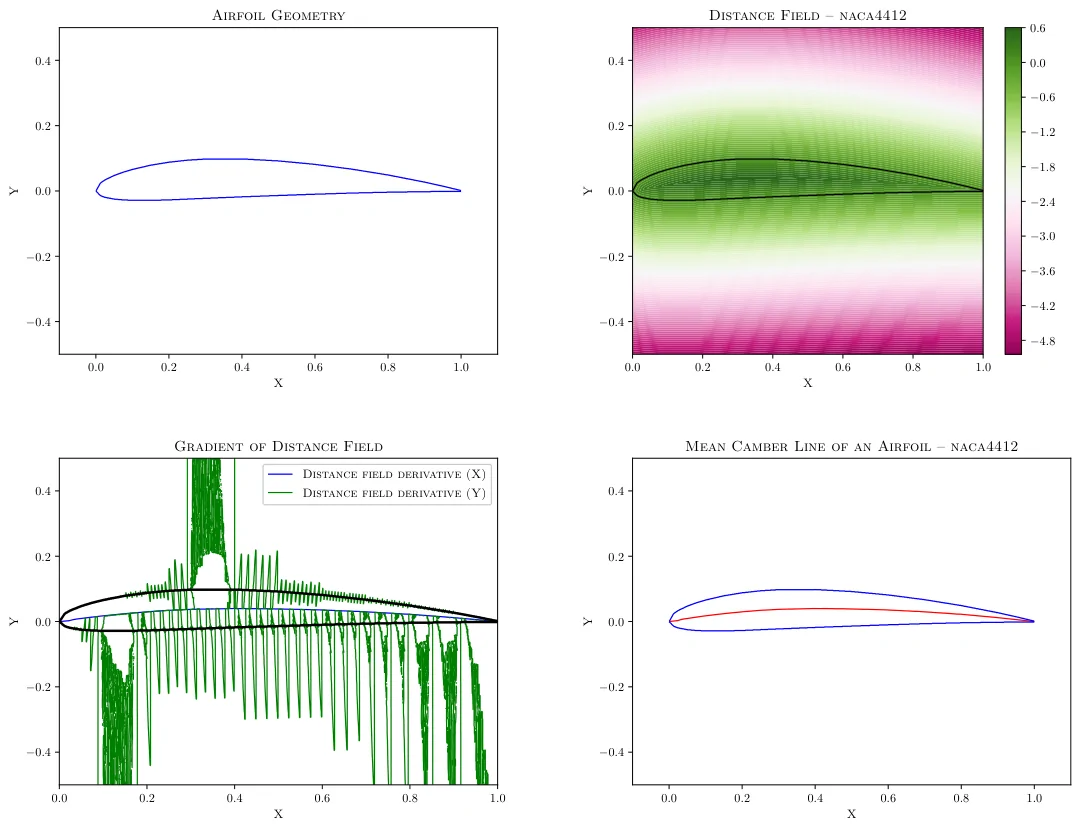
Grid-based mean camber line workflow showing the airfoil boundary, distance-like field, derivative/ridge response, and extracted curve.

The mean camber line example uses a different computational representation. Rather than editing a surface mesh, the script projects an airfoil boundary onto a Cartesian grid, computes a signed distance field, evaluates gradient and Hessian information, groups field samples by their grid-column index, and extracts a ridge-like interior response. The workflow follows the distance-field-driven approach of [22] and combines geometry input, grid construction, scalar-field computation, finite differences, smoothing, resampling, and curve-network output. Listing 8 gives the executable flow, and Figure 12 shows its principal stages.

**Listing 8:** Flow-style *tgLang* script for distance-field-driven mean camber line extraction.

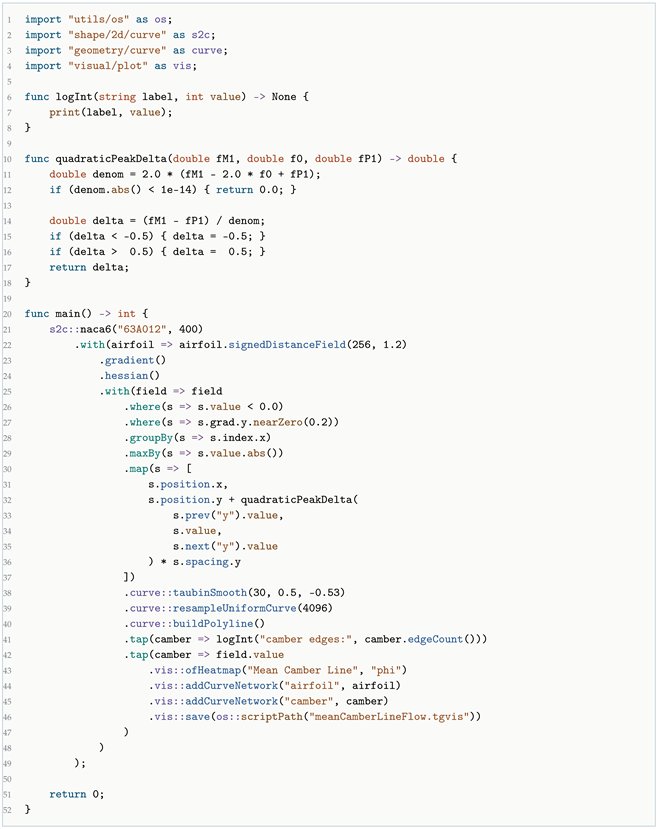



This case demonstrates *tgLang* as a coordination language for auxiliary representations such as distance fields: the same source file describes input handling, grid setup, field operations, and curve extraction.

### 4.3. Point-Cloud Workflows

#### Voxel Downsampling

**Figure 13:**
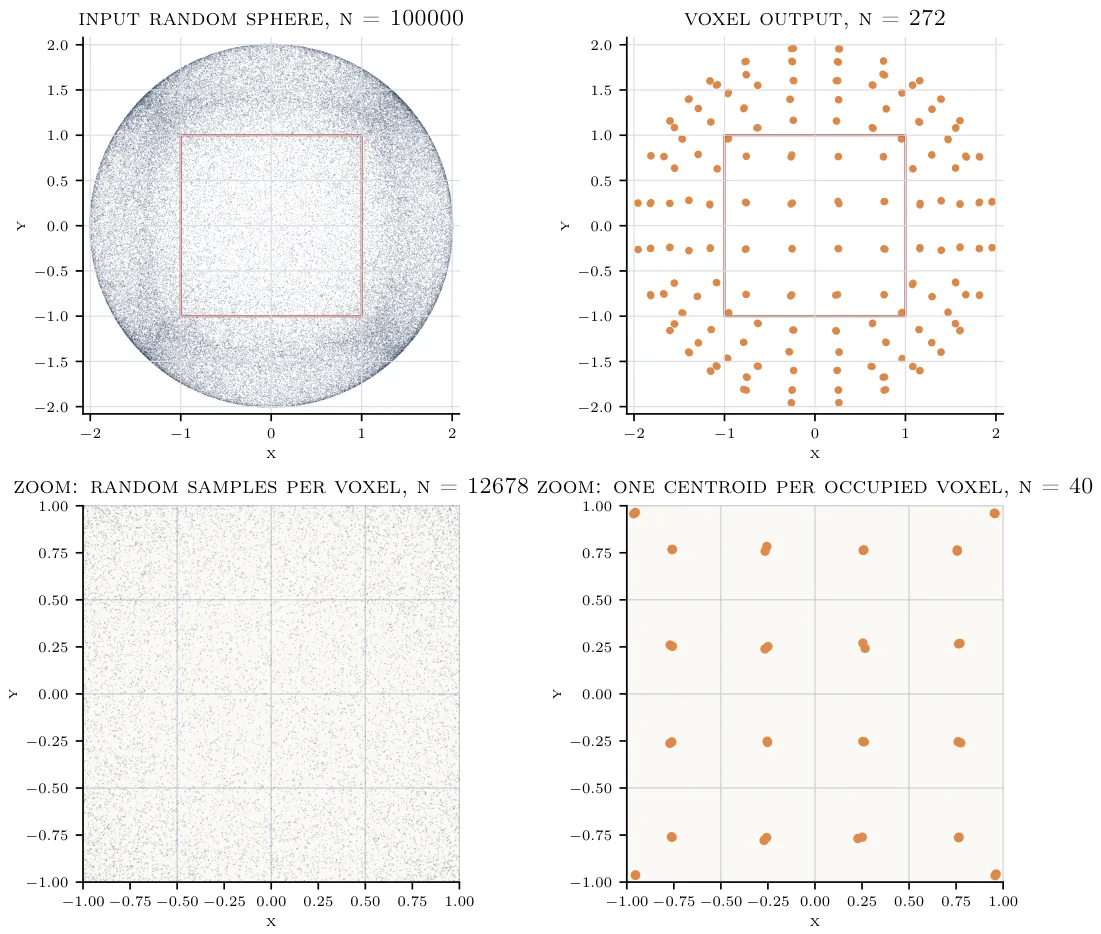
Voxel-grid downsampling of a randomly sampled sphere. The shared zoom window contrasts dense random input samples with the voxel-centroid output; in the selected window, 12,678 samples reduce to 40 occupied-cell representatives.

Point clouds occur frequently in scanning, reverse engineering, and preprocessing pipelines. Voxel downsampling is representative because it is easy to specify but still touches several programming concerns: assigning points to cells, grouping by occupied voxel, reducing each group to a centroid, converting the centroids back to a point cloud, and visualizing the before/after result. In *tgLang*, the flow form expresses these steps through a generic groupBy–map pipeline rather than a dedicated downsampling keyword. Listing 9 gives the flow, and Figure 13 shows the input and voxel-centroid output.

**Listing 9:** Flow-style *tgLang* script for voxel point-cloud downsampling.

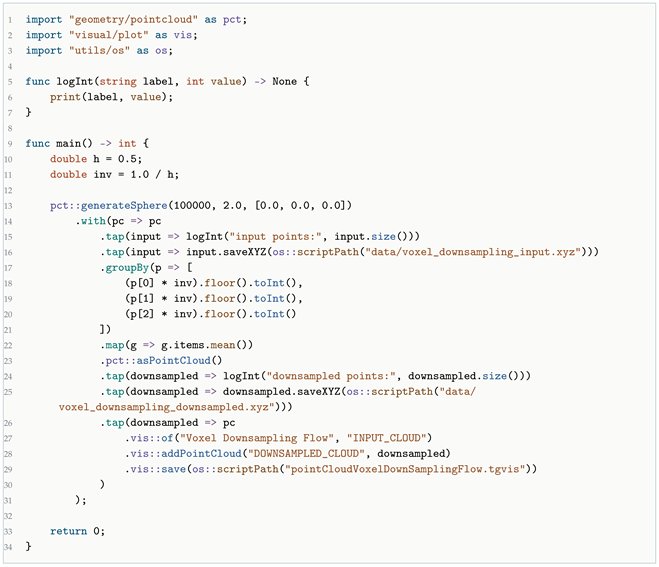



### 4.4. Image and Image-Stack Workflows

**Figure 14:**
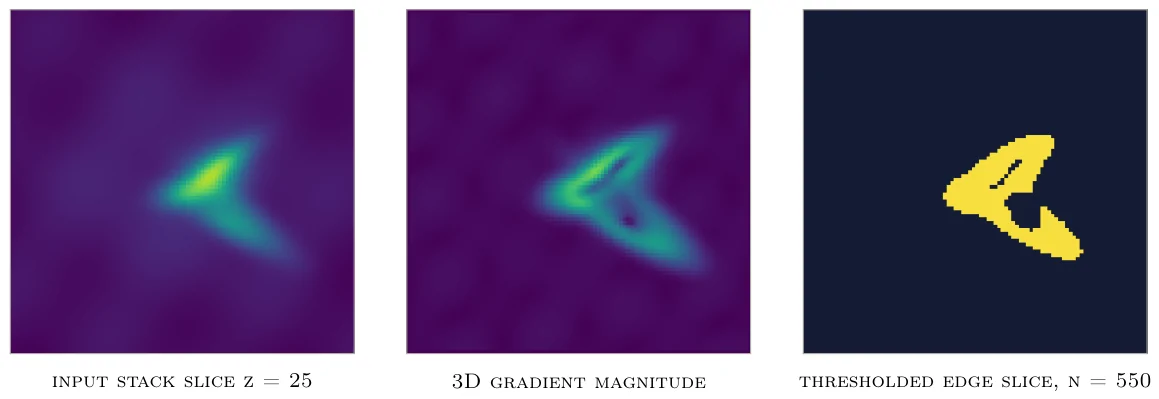
Three-dimensional edge detection on a synthetic image stack. The workflow keeps the central-difference stencil visible in the source program, computes a stack-wide gradient-magnitude volume from the voxel stream, and thresholds the result into an edge mask.

**Figure 15:**
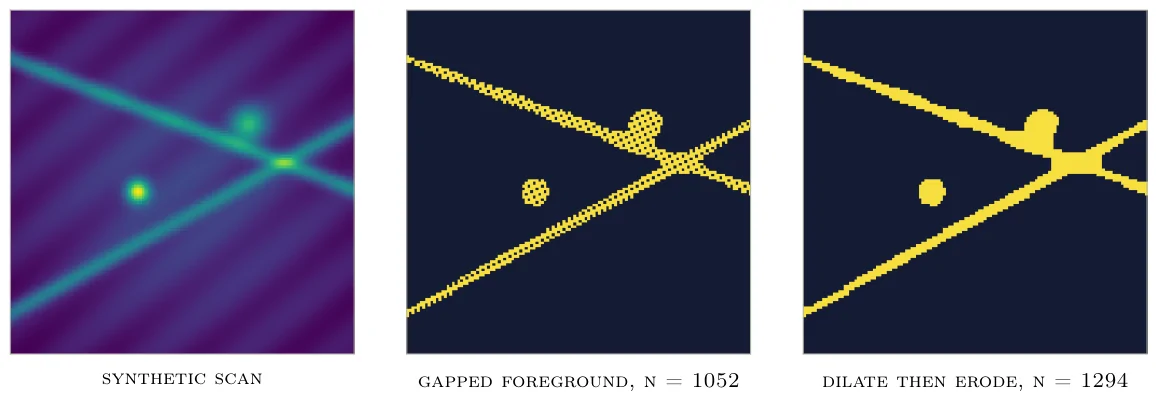
Morphological closing workflow on a synthetic binary mask. The script explicitly applies dilation followed by erosion, turning a deliberately gapped foreground mask into a repaired binary region.

**Figure 16:**
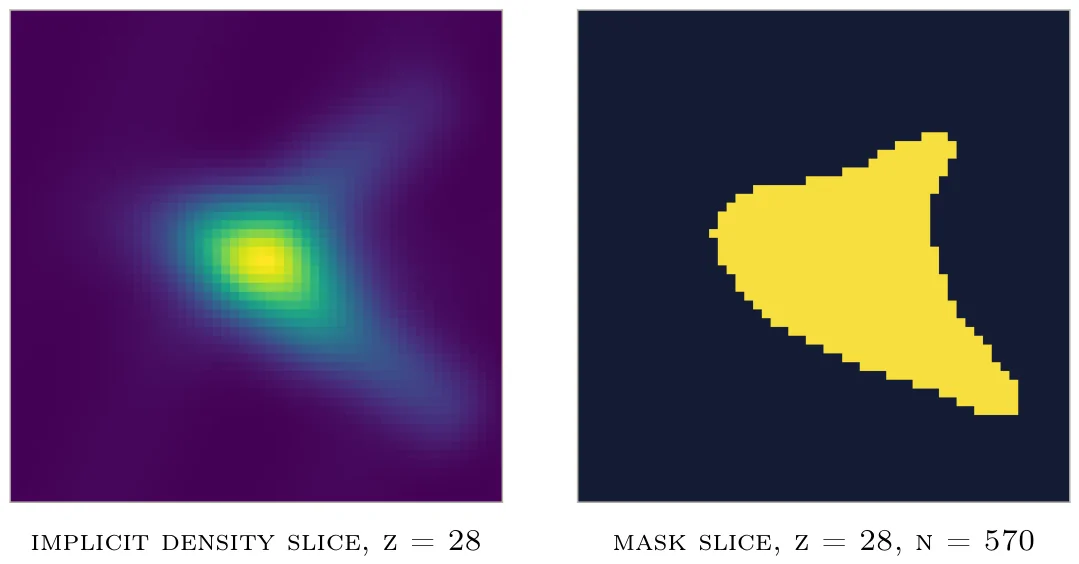
Implicit-field voxelization on a 56×56×56 image stack. The source program defines the branching density from capsule distances, lobes, and a small sinusoidal perturbation, and it then thresholds the field into a binary image3d. The panels show the mid-plane slice at zero-based index z=28.

**Figure 17:**
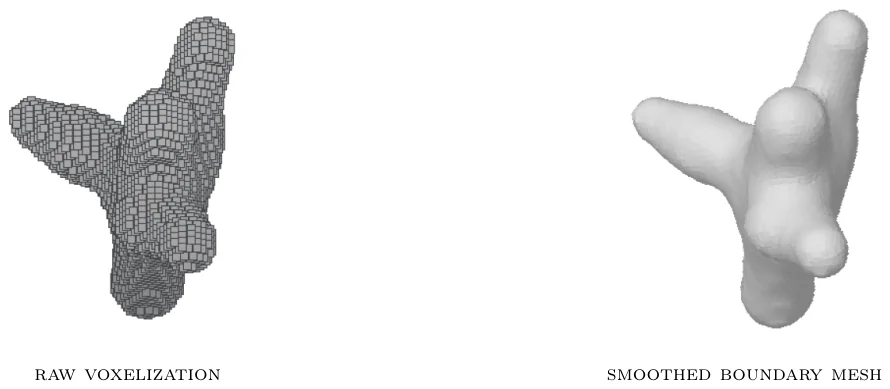
Implicit image-stack workflow from voxels to geometry. The left panel shows the raw binary voxelization of the 56×56×56 implicit field; the right panel shows the extracted and smoothed boundary triangle mesh with 11,664 faces.

The image examples use the same flow model on raster and stack data. The image2d type stores dense scalar images and exposes pixel-level streams, morphology operations, heat-map plotting, and conversion from flow-computed pixel arrays. The image3d type represents an image stack: slices can be accessed individually, while voxel streams and projection methods support volumetric image-processing examples. The listings import image/synthetic for explicitly named data constructors, such as synthetic2dScan and synthetic3dVolume so that the manuscript is self-contained. The visible code focuses on the image-processing flow itself and uses the same flow operators as the geometry examples, without introducing a separate mini-language for pixels or voxels. Listings 10–12 present the corresponding algorithms; Figure 14, Figure 15, Figure 16 and Figure 17 show their outputs in the same order.

**Listing 10:** Flow-style *tgLang* script for three-dimensional edge detection on a synthetic image stack.

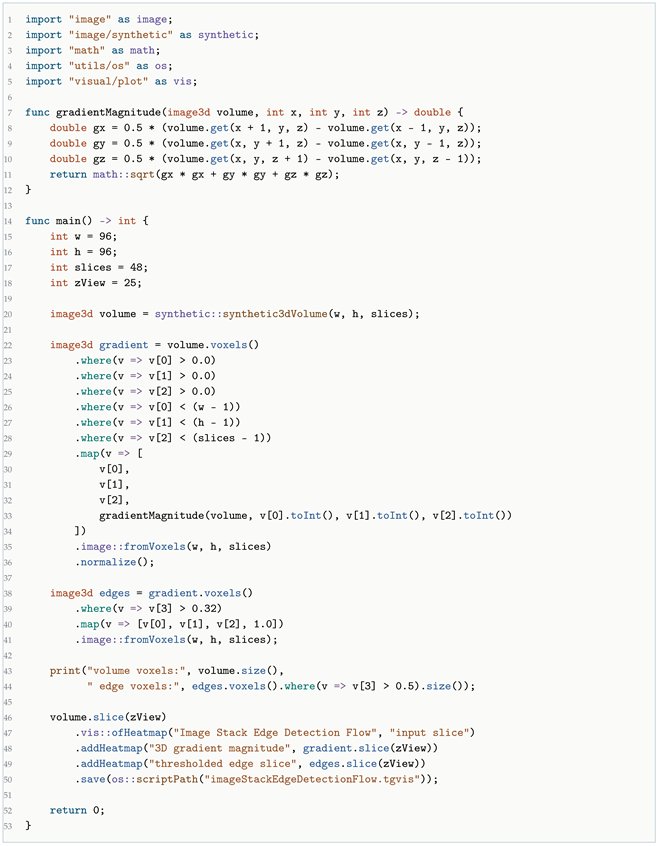



**Listing 11:** Flow-style *tgLang* script for morphological closing on a synthetic binary mask.

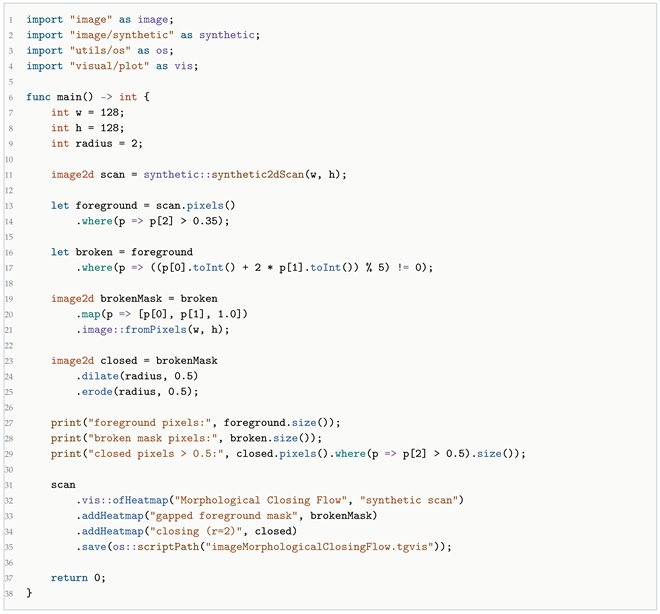



**Listing 12:** Algorithmic excerpt for implicit-field evaluation, voxelization, boundary-surface extraction, and mesh smoothing.

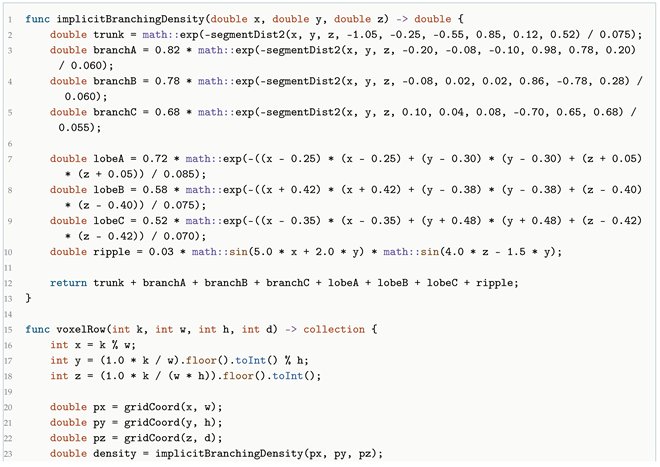



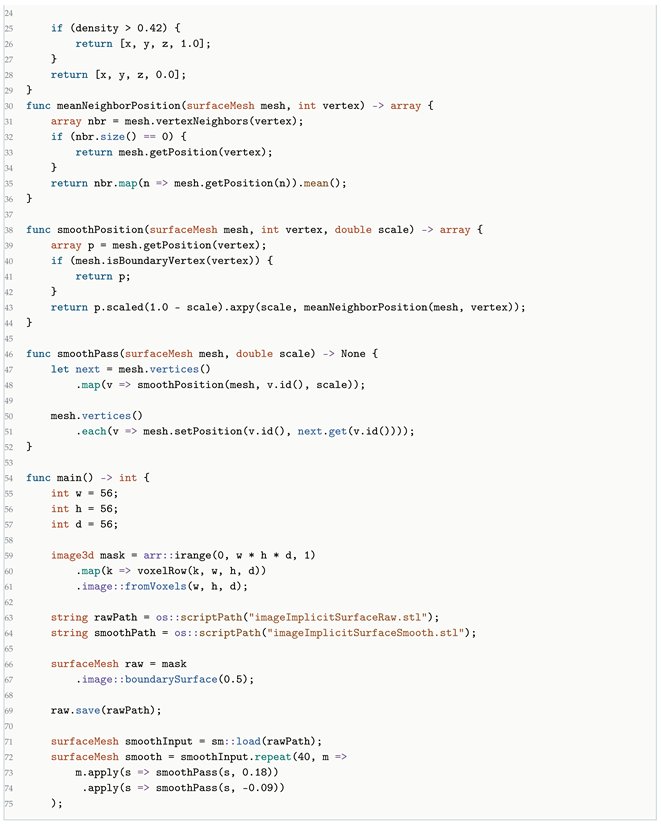



The implicit-volume excerpt deliberately retains both voxelRow, which maps a linear grid index to a thresholded voxel, and the flow from voxel rows to image3d and then to a boundary surfaceMesh. Only the coordinate-distance helper and final diagnostics/plotting code are omitted; the complete executable source is supplied through the reproduction page.

### 4.5. Curve-Network Workflows

**Figure 18:**
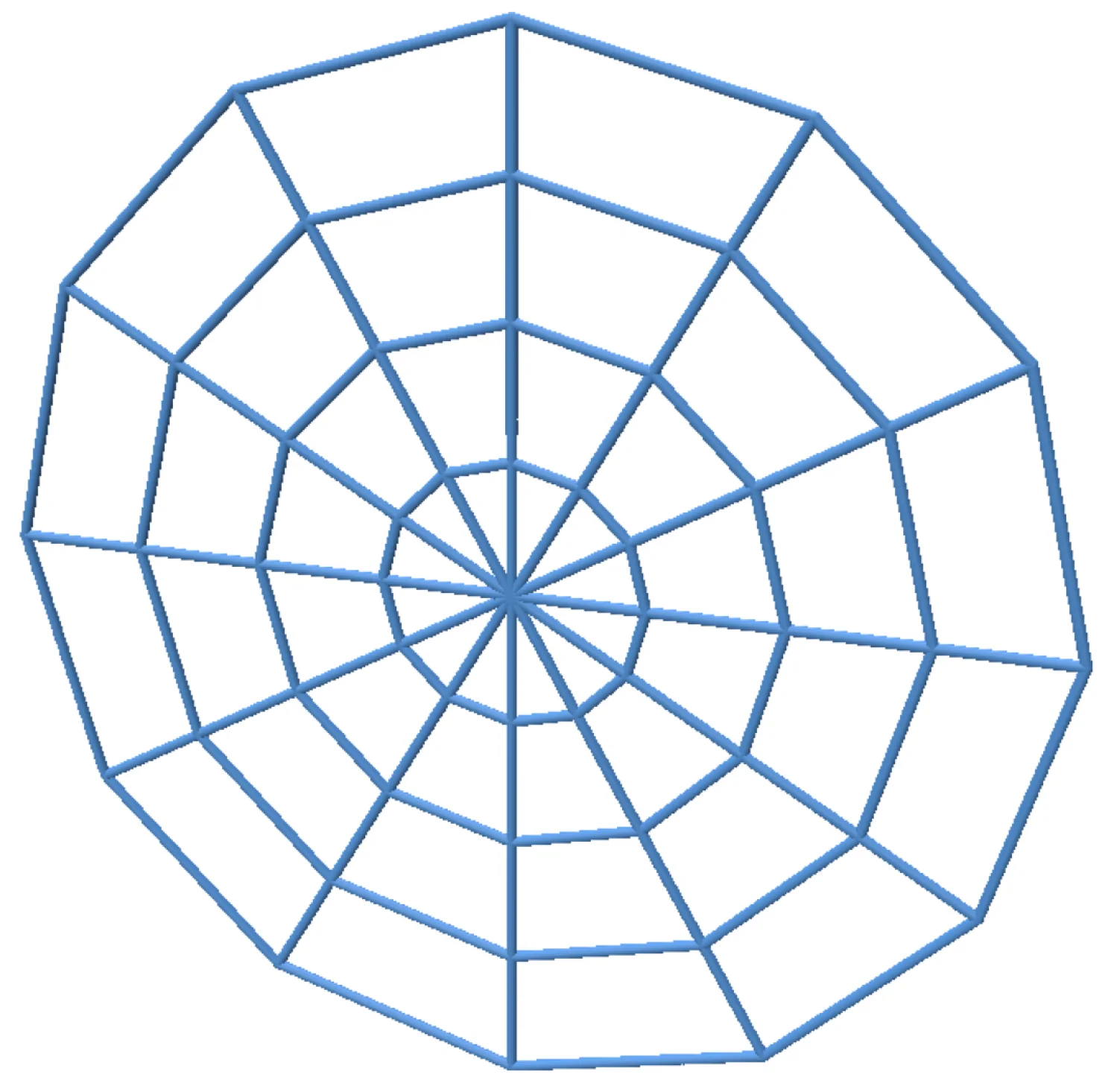
Procedurally generated polar web assembled from filtered concentric-ring and radial-spoke curve networks.

Curve networks model embedded polylines or graph-like structures, which are useful for skeletons, intersection curves, and extracted features. Listing 13 generates concentric rings and radial spokes as collections of curve networks, filters rings by geometric length, reports the resulting collection sizes, and sends both collections to the visualization module. The example is retained in full because it demonstrates that the same flow operators used for mesh, point-cloud, and image values also compose graph-like geometric values. Figure 18 shows the generated network.

**Listing 13:** Flow-style *tgLang* script for procedural polar-web curve networks.

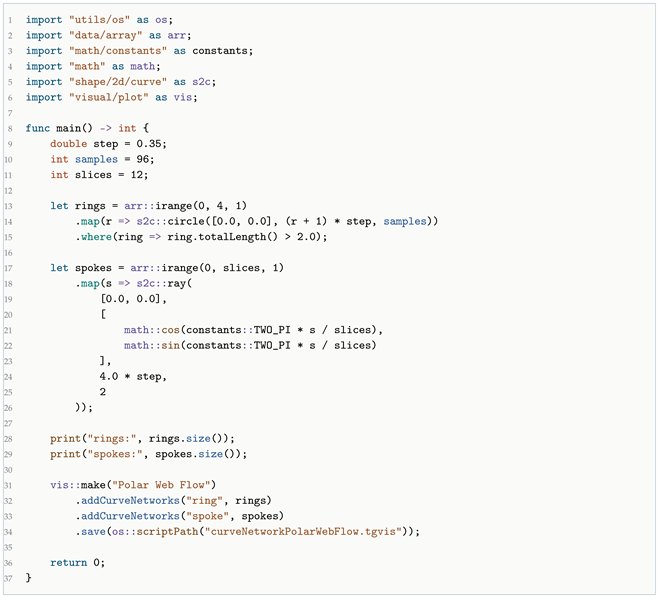



Taken together, the examples show that the language spans multiple computational representations. The same source-level model is used for mutable surface meshes, unstructured point sets, structured grids, scalar fields, curve networks, images, and image stacks. The newer flow examples also show that the concise notation is not a separate mini-language: it composes ordinary methods, module functions, lambdas, internal iteration values, and plotting or console side effects inside the same typed compiler pipeline.

### 4.6. Parallel Workflow Evaluation

Parallelism is evaluated separately from the representation workflows because it tests the execution model rather than a new geometry or imaging type. The evaluated workloads are paired serial and parallel flow programs over point-cloud, image-stack, and image-tile data. Each pair uses the same input size and output representation, so the comparison isolates the effect of deterministic worker execution on the surrounding algorithm.

**Figure 19:**
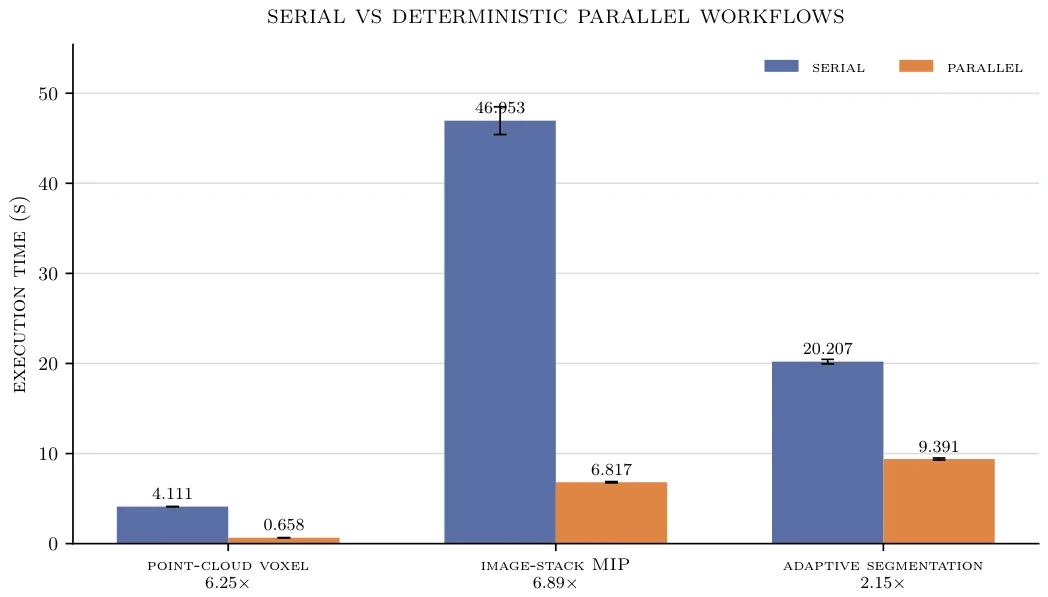
Mean serial and deterministic-parallel execution times for representative flow workflows over ten fresh-process measurements. Error bars show one sample standard deviation.

The comparison reports execution time and speedup for the serial and parallel versions of these workflows. One unreported warm-up precedes ten measured fresh-process executions of each version; each execution mean is reported with its sample standard deviation and two-sided 95% Student-*t* interval, while speedup is the ratio of the serial and parallel execution-time means. This keeps parallelism in the paper as an experimental result: cloned worker runtimes, fixed-order result collection, and reproducible outputs are evaluated on complete geometry and imaging workloads rather than on synthetic scalar loops alone. For the image workflows, the serial comparison scripts construct the same synthetic scan or volume through ordinary tgLang flow maps, so the reported speedup measures deterministic worker execution rather than replacement of source-level work by a C++ data constructor. Figure 19 visualizes the timing comparison, and Table 9 reports the numerical values.

Listing 14 shows the complete source-level form for one evaluated parallel imaging workflow. The parallel operation is an ordinary module-qualified flow call: the surrounding program still defines the mapped worker function and performs grouping, image conversion, mask construction, and visualization in the same typed source. The point-cloud workload uses parallel::map followed by deterministic keyed reduction, while the image-stack workload uses parallel::map for voxel generation before typed volume construction and projection. Their complete sources remain in the artifact, avoiding two near-duplicate full listings while preserving all three evaluated programs and timing results.

**Listing 14:** Parallel adaptive image-tile segmentation. The source keeps image generation, tile grouping, mask construction, and visualization in one typed program.

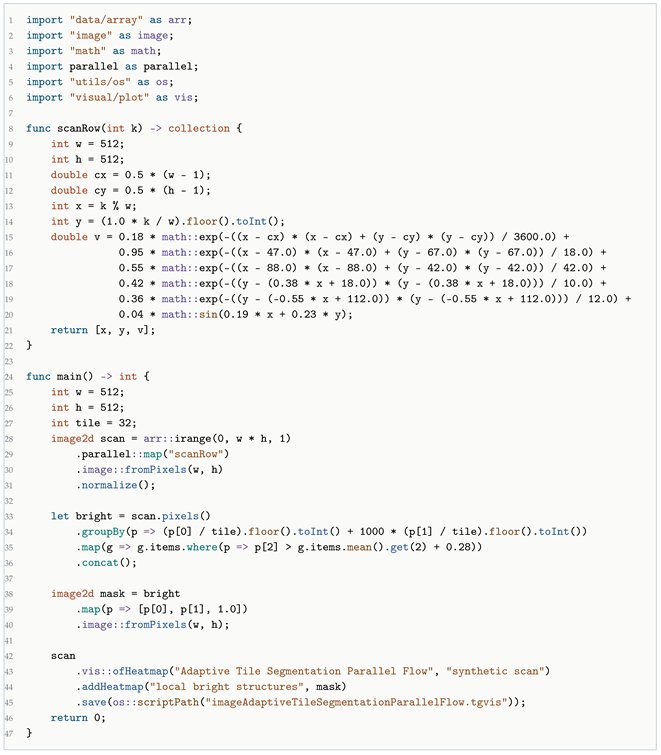



The point-cloud and image-stack mappings reach 6.25× and 6.89× speedup, respectively, because each worker performs enough arithmetic to amortize runtime cloning and ordered result collection. Adaptive segmentation reaches 2.15×: its worker products are smaller, but tile grouping, collection assembly, and final mask construction remain serial. Workers are threads with worker-local runtimes rather than remote processes, so there is no network serialization. The relevant fixed costs are runtime cloning, worker-local allocation, deterministic merging, and, when tracing collection is enabled, adoption of the returned object graph into the parent heap.

### 4.7. Runtime Performance

Runtime measurements apply to an implementation, not to a grammar. The results below therefore characterize the current tgVM backend and its native runtime kernels; they are not an intrinsic performance limit of tgLang programs. A native C++ or accelerator backend preserving the same source semantics would execute the same programs with a different cost model. Accordingly, this section reports reproducible absolute workflow timings and parallel speedups rather than treating a comparison between tgVM bytecode and a selected library kernel as a comparison between two source languages.

The workflows were run through tgVM, the reference execution path for the current implementation, using the arm64 macOS build of *tgLang* 1.4.0 identified in the benchmark manifest. Measurements were collected on an idle Apple Mac mini with an Apple M4 processor (10 logical CPUs), 16 GiB unified memory, and macOS 26.5.2. For each workflow, one unreported warm-up was followed by ten measured executions, each in a fresh process. The runner retains every raw observation and reports the arithmetic mean, sample standard deviation, and a two-sided 95% confidence interval using Student’s *t* distribution with nine degrees of freedom. The command-line execution timer excludes process startup but includes source loading, bytecode execution, native dispatch, algorithm work, and requested output generation; an independent operating-system measurement records peak resident memory. All workflow runs use –threads=10. These are end-to-end application measurements rather than isolated opcode microbenchmarks; they characterize the evaluated implementation but are not presented as evidence of general superiority over specialized native or vectorized libraries. Table 10 reports the complete workflow measurements.

The workflow spread is intentional. Curve construction and two-dimensional morphology expose fixed process and VM costs, whereas image-stack differentiation and implicit-surface extraction materialize large dense intermediate values. Point-cloud downsampling is implemented through generic groupBy and map, rather than a hidden voxel kernel, and therefore exposes the present cost of bytecode-level grouping. Hole detection, smoothing, and remeshing spend more of their execution in native topology and geometry operations. Table 11 separately measures tracing-collector cost on the allocation-heavy parallel cases.

Enabling tracing collection increased both execution time and peak RSS in these short, allocation-heavy programs. Collection introduces tracing, promotion, and remembered-set costs, while the maximum live set remains large enough that few native payloads can be released early. This negative result supports the default-off policy for fresh-process batch workflows. It does not imply unbounded leakage between runs: runtime teardown releases every tracked object. Long-lived REPL or server sessions can enable –garbageCollect to reclaim unreachable temporaries during execution. Table 12 reports the corresponding fixed-input determinism checks across worker counts.

The three tested map/group/reduction workflows produced identical canonical files at 1, 2, 4, and 10 workers. This is a bounded empirical guarantee: deterministic partitioning, per-worker state, and ordered collection constrain the supported parallel combinators. The claim does not cover arbitrary topology-changing mutation, unordered external native libraries, or bit-identical floating-point output across processor architectures.

## 5. Discussion

The results support the central premise of *tgLang*: geometry and imaging workflows benefit when domain entities, execution rules, diagnostics, and visualization are expressed in one typed source language. The workflow scripts are not only shorter versions of C++ or Python programs. They expose the algorithmic objects that matter: selected mesh edges, connected components, scalar-field samples, voxel groups, image pixels, image-stack voxels, curve networks, and plots. Once these objects are part of the language/runtime model, the compiler can preserve context across filters, maps, grouping operations, and side-effect taps instead of treating every step as an opaque library call.

This design changes where complexity is placed. Conventional library code gives the programmer direct access to mature kernels but also exposes traversal conventions, ownership rules, conversion code, and backend-specific structure. *tgLang* moves these recurring concerns into typed domain values and runtime modules. The resulting source code can describe the workflow at the level used in papers and algorithm sketches: select candidate hole edges, group field samples by grid column, reduce voxel groups to representatives, apply morphology to a mask, or project an image stack. The runtime remains responsible for efficient data structures and robust kernels, but the program that coordinates them is explicit, runtime-validated, and reproducible.

The flow layer is the clearest example of this tradeoff. Operators such as where, map, groupBy, maxBy, components, tap, and module-qualified calls are deliberately generic. They are not aliases for one mesh algorithm or one image routine; they are compiler-visible coordination patterns. Their effectiveness depends on internal runtime values carrying enough domain context: selected edges must retain their parent mesh, field samples must retain grid coordinates and derivative values, and pixels or voxels must retain raster positions. When that context is present, concise source code remains semantically rich. When it is thin, a generic operator becomes an ordinary loop and performance suffers.

The timing results characterize the present tgVM implementation. The point-cloud downsampling example is intentionally expressed through generic groupBy and map rather than hidden behind a specialized voxel-downsampling kernel. This strengthens the language argument because the same grouping abstraction also applies to scalar-field samples and image data, while identifying specialized internal data structures, caching, and parallel grouping as implementation opportunities that do not require source-language changes.

The current VM-centered implementation is therefore best understood as a reference execution system for a domain-specific source language. tgVM defines the observable semantics, deterministic parallel behavior, source-level diagnostics, module dispatch, and object lifetime rules. Expensive operations are already delegated to native kernels, and additional native-code targets can be added without changing the source model. This makes the language useful today for reproducible scripts and paper workflows, while leaving a clear path toward standalone executables or shared-library deployment for performance-critical use.

Several engineering directions follow directly from the examples. Generic grouping and stream operations should be optimized for large point-cloud, field-sample, and image-stack workloads. Runtime modules should continue to grow with stronger validation and diagnostics for non-manifold meshes, empty selections, invalid masks, inconsistent metadata, and failed conversions. Because tgLang is the language and tgVM is only its present reference backend, performance work does not require a new grammar or rewritten applications. A future bytecode-to-C++ backend could produce native CPU executables or libraries from the same programs, while suitable parallel flow regions could be lowered to GPU kernels under the same type and ordering rules. Finally, a broader benchmark suite and user study would complement the technical evaluation by measuring prototyping time, error rates, and maintainability in comparison with conventional Python and C++ library workflows.

The present adoption barriers are concrete. tgLang has a much smaller module ecosystem than Python or C++; native extensions require a runtime rebuild because there is no stable binary plugin ABI; the downloadable reproduction image targets x86-64 Linux, while native development is currently concentrated on Linux and macOS; and browser execution does not expose every native geometry kernel through WebAssembly, so the public paper route uses the managed server backend. The compiler/runtime implementation is proprietary, although the evaluated workflow sources are available. Users must also understand the underlying domain representations: concise syntax cannot make a non-manifold edit or ill-posed image operation valid. No claim about learning time or programmer productivity is made; a controlled study with users independent of the language developers remains future work.

## 6. Conclusions

The central claim of this paper is that geometry-processing and computational-imaging workflows benefit from a language layer that makes domain representations first-class, explicitly typed values, without forcing the corresponding optimized kernels to be rewritten inside that language. *tgLang* realizes this separation by treating meshes, point clouds, grids, curve networks, images, and image stacks as ordinary typed values and expressions, while dispatching their expensive topological, geometric, and raster operations to native runtime kernels. The result is a compact source-level notation that remains executable, reproducible, and inspectable.

The language design is deliberately conservative in its surface syntax but specific in its execution semantics. Manifest types record representation assumptions and are validated at the VM/native boundary; modules and extendModule provide a uniform namespace model for both built-in and experimental operations; and deterministic parfor and flow-oriented operators such as with, where, groupBy, tap, and components give recurring workflow patterns a predictable, compiler-visible form. These constructs are not merely syntactic sugar: they affect where diagnostics can be localized, how parallel results can be reproduced, and how exploratory code can mature into shared modules without being reimplemented in a host language.

The workflow evaluation supports this claim across several representative domains. Feature-edge hole detection combines connectivity queries, curvature filtering, and cycle extraction in a single typed flow. Mean camber line extraction coordinates airfoil geometry, signed distance fields, gradient and Hessian tests, grouping, and curve extraction. Voxel downsampling, curve-network generation, surface smoothing and remeshing, image-stack edge detection, explicit morphology, and implicit-field surface extraction demonstrate that the same programming model spans unstructured samples, graph-like curves, scalar fields, mutable meshes, dense images, image stacks, and extracted surface meshes. The parallel timing results further show that deterministic worker execution is practical on complete workloads rather than only on synthetic scalar loops.

The implementation now creates several clear engineering directions. Generic flow operators such as groupBy should be optimized for large point-cloud and image-stack streams, because they are central to keeping workflows representation-generic. The standard geometry and imaging modules should continue to grow with robustness checks and richer diagnostics so that failures in non-manifold meshes, empty selections, invalid image masks, or inconsistent metadata are reported at the source-program level. A future bytecode-to-C++ pipeline is the natural native deployment path for workflows that begin under tgVM and later require standalone executables or shared libraries; accelerator-suitable flow regions could likewise target GPU runtimes. Both routes would retain the tgLang grammar and source program. A larger benchmark suite and user study would complement the technical evaluation by measuring prototyping time, error rates, and maintainability.

In short, *tgLang* treats workflow expression as a first-class research problem. The workflows in this paper show that typed, domain-aware source programs can make representation choices explicit, keep multi-stage algorithms inspectable, and reproduce results across mesh, grid, point-cloud, curve, and image domains.

## Figures and Tables

**Table 1:** Comparison between a conventional library-oriented workflow and the language-level model used by *tgLang*.

Concern	Typical Library-Level Handling	Language-Level Handling in *tgLang*
Domain intent	Inferred from library classes, traversal idioms, and documentation	Declared through first-class types such as surfaceMesh, pointCloud, curveNetwork, image2d, and image3d
Algorithm structure	Interleaved with traversal code, conversions, ownership handling, and backend calls	Expressed as typed source code in which domain operations are available through modules and methods
Reproducibility	Relies on host-language behavior, library conventions, and user discipline	Deterministic execution rules are specified by the runtime, including supported parallel combinators
Diagnostics	Failures may surface through template errors, runtime assertions, or wrapper-layer messages	Errors can be associated with typed source constructs and bytecode locations
Deployment	Exploratory scripts and production code can become separate implementations	One source program can be run locally, in server-backed browser sessions, or in reproducible batch scripts; tgVM is the current backend rather than a source-language restriction
Extensibility	New operations are commonly introduced through external APIs or project-specific wrappers	Modules and extensions use the same namespace and signature model as built-in operations

**Table 2:** Scope comparison with adjacent libraries and domain-specific systems. Entries describe facilities native to the stated programming model; functionality supplied by unrestricted host-language or external-library code is identified separately. “Subset” denotes a bounded facility rather than a system-wide guarantee.

System	Primary Representation	Mutable Topology	Image Stacks	Composition Model	Execution and Extension Boundary
C++ geometry libraries	Library-specific meshes and arrays	Yes	Separate libraries	Host-language calls	Native CPU kernels; templates and C++ APIs
Python/Open3D	Meshes, point clouds, images	Limited by API	Limited	Python calls	Native library kernels through Python bindings
Liszt	Mesh topology and fields	No	No	Mesh kernels	Compiled parallel mesh/PDE kernels
Ebb	Relational geometric domains	No in the reported implementation	No	Data-parallel kernels	CPU/GPU runtime; Lua orchestration and external-library calls
Simit	Hypergraphs and tensors	No in the DSL; C++ between executions	No	Tensor/index expressions	Compiled CPU/GPU simulation; C++ interoperability
Halide	Functional images	No	Yes	Algorithm/ schedule pipelines	Optimizing CPU/GPU compiler; external functions
Taichi	Dense/sparse fields and particles	Not general mesh edits	Yes	Imperative kernels	JIT/AOT CPU/GPU compilation; Python/C++ integration
MeshTaichi	Mesh relations and attributes	No	No	Mesh kernels	CPU/GPU compiler with relation/layout analysis
I♥MESH	Symbolic mesh expressions	No in the reported language scope	No	Symbolic expressions	Translation to host-language backends; procedural algorithms remain in the host
*tgLang*	Meshes, points, curves, grids, images	Yes	Yes	Imperative and flow pipelines	Current: tgVM plus native kernels; grammar and programs are backend-independent

**Table 3:** Representative built-in types in *tgLang*.

Category	Types and Role
Primitive	Scalar and basic values: int, double, bool, string, none
Collections	Homogeneous typed arrays via array<dtype, rank|shape>; heterogeneous values via collection; associative data via table
Geometry	Built-in representations including pointCloud, curveNetwork, surfaceMesh, volumeMesh, lineGrid, squareGrid, cubeGrid, and fdmGrid<dim>
Images and image stacks	Dense image values via image2d and stack/volume image values via image3d; image methods expose pixels, voxels, slices, morphology, and projection operations
Input/output and diagnostics	Script-visible I/O, visualization, and failure values: ifile, ofile, plot, error
Extensibility	Lightweight record-like values defined with special, mainly for packaging reusable domain concepts

**Table 4:** Boundary between compilation checks and runtime validation in *tgLang*.

Concern	Compilation	Execution in tgVM/Runtime
Declarations and names	Parses explicit variable, parameter, and return annotations; records local representation hints; resolves lexical names, imports, module aliases, and user-defined declarations	Constructs default or initialized values and reports missing runtime registrations
Functions and methods	Records source-level signatures and rejects duplicate declarations and structurally invalid calls	Selects an exact native overload by receiver and argument types; controlled any fallbacks are limited to generic sinks and containers; no arbitrary cross-representation coercion is performed
Numeric values	Distinguishes integer and floating-point literals in emitted bytecode	Mixed arithmetic promotes int operands to double; native function overloads otherwise require their registered argument types unless an overload explicitly accepts another numeric type
Arrays	Parses element-type, rank, and shape annotations and emits typed-array construction bytecode	Validates element dtype, rank, bounds, and operation-specific shape constraints; dimensions depending on input data are necessarily dynamic
Domain representations	Makes surfaceMesh, pointCloud, image2d, image3d, grids, and curves distinct source annotations	Validates constructors, receiver types, property lengths, image dimensions, topology preconditions, and geometric validity where required by an operation
Flow values	Binds compiler-known element context for mesh entities and lowers lambdas to bytecode loops	Internal selections, groups, samples, pixels, and voxels remain runtime-only values and are checked by the consuming flow operator
Parallel constructs	Rejects unsupported control flow and direct writes to captured non-local variables in parfor bodies	Validates iterable/range values and executes supported collection with worker-local runtimes and ordered result assembly

**Table 5:** Flow operators and compiler-level behavior in the current *tgLang* implementation.

Operator Form	Meaning
x.with(a => expr)	Binds the current value x to the lambda parameter a and evaluates expr. The result of expr becomes the result of the whole flow. This is used to keep an earlier value, such as an input mesh, available while a derived stream is processed.
x.tap(a => expr)	Executes expr for diagnostics, plotting, writing, or other side effects, then returns the original pipeline value. The lambda receives a cloned snapshot of the receiver, so the tap is not allowed to mutate the value continuing through the pipeline. State-changing transformations must be expressed with map, module calls, or ordinary methods.
x.where(a => pred)	Filters a collection-like or stream-like value by a predicate over its current element. For geometry-derived streams, the element can carry domain context, such as an edge together with its parent mesh.
x.all(a => pred)	Tests whether every current element satisfies a predicate. This is useful for local topological conditions, for example checking whether all vertices incident to a selected edge satisfy a valence constraint.
x.map(a => expr)	Applies a transformation to each current element and returns the transformed collection. It is the flow operator for changing pipeline state element-wise.
x.each(a => expr)	Evaluates expr for each current element when the algorithm intentionally performs mutation or incremental construction, for example applying stored displacements to mesh vertices.
x.repeat(n, a => expr)	Repeatedly evaluates a state-transforming lambda n times, feeding the previous result into the next iteration. This expresses fixed-iteration workflows such as smoothing without exposing loop counters when they are not algorithmically important.
x.apply(a => expr)	Evaluates a side-effecting lambda and returns the original receiver. Unlike tap, this form is intended for deliberate stateful application rather than diagnostic observation.
x.groupBy(a => key)	Partitions elements by a key computed in a lambda. This supports generic reductions such as voxel grouping for point clouds without introducing a point-cloud-specific downsampling keyword.
x.maxBy(a => score)	Selects the element with the maximal score from a group or grouped stream. In the mean-camber example, this chooses one interior distance-field sample per grid column.
x.components()	Converts a selected adjacency-bearing stream into connected components. For selected mesh edges, this groups candidate edges into connected edge components before cycle filtering.
x.concat()	Concatenates a collection of compatible stream values or arrays by one level, preserving tuple-like array elements. This is used when independent incoming streams, such as sampled surfaces and synthetic outliers, must be joined before later flow stages.
x.mod::fn(...)	Invokes a module function as a pipeline stage, with the current receiver supplied as the first argument. This lets reusable module functions, such as curve smoothing or plot construction, participate in a chain without redefining them as methods.

**Table 6:** Selected TGBC instructions used by the tgVM execution path.

Opcode	Role
PushConst i	Pushes a constant from the constant pool.
LoadLocal i	Loads a local variable onto the operand stack.
StoreLocal i	Stores the stack top into a local variable.
LoadGlobal i/StoreGlobal i	Reads or writes a value in global/module scope.
ImportModule i j	Imports a module and binds an alias.
LoadModuleVar i/StoreModuleVar i	Reads or writes a module-scoped variable.
CallFunction i n	Calls a function by name and arity, including VM helper functions used by lowered flow operators.
CallModuleFunction m f n	Calls a module-scoped function; module-qualified pipeline calls lower to this opcode with the receiver supplied as the first argument.
CallMethod i n	Invokes a type method on a receiver.
FieldGet i/FieldSet i	Reads or writes a field on a user-defined record object.
IndexGet n/IndexSet n	Indexed access for arrays, collections, and tables.
Jump a/JumpIfFalse a	Unconditional and conditional control-flow transfer.
Return	Returns from the current function.
Try a b, Throw i b, EndTry	Structured exception handling.
BuildVector a b/BuildMatrix a b	Constructs vector, collection, and matrix literals.
BuildArray r n/BuildTable n	Constructs typed arrays and table literals.
BinaryOp op/UnaryOp op	Arithmetic and logical operations.
Pop/Print	Discards a value or prints values for statement-level output.
ParForRange a b c/ ParForRangeInclusive a b c	Deterministic parallel range loops.
IndexGetLinear	Fast linear index access for array-like values.

**Table 7:** Systematic coverage of the representative workflow set.

Workflow	Representation	Language/Runtime Feature	Execution Mode	Input
Hole detection	Surface mesh, edge components, curves	Contextual filters, connectivity, native mesh queries	Read-only topology	Real triangulated meshes
Taubin smoothing	Surface mesh	Neighborhood reductions and repeated application	Vertex mutation, serial	Controlled noisy sphere
Longest-edge subdivision	Surface mesh	Queue-like selection and native topology edits	Topology mutation, serial	Coarse demonstration mesh
Isotropic remeshing	Surface mesh	Split/collapse/flip, smoothing, projection	Topology mutation, serial	Thingi10k mesh
Mean camber line	Curve, grid, scalar field	Derivatives, grouping, maxima, curve conversion	Read-only flow, serial	Analytic NACA airfoil
Voxel downsampling	Point cloud and groups	Generic keying, grouping, centroid reduction	Serial and parallel variants	Controlled sampled sphere
Stack edge detection	image3d	Explicit finite differences over voxels	Serial voxel flow	Synthetic volume
Morphological closing	image2d	Pixel filtering, dilation, erosion	Native image kernels	Synthetic gapped mask
Implicit surface	image3d, surface mesh	Implicit evaluation, voxelization, boundary extraction	Serial flow and mesh mutation	Synthetic implicit object
Polar web	Curve networks	Mapping, filtering, reductions, plotting	Read-only flow	Procedural curves

**Table 8:** Structural source metrics for the complete representative *tgLang* workflows.

Workflow	SLOC	Functions	Imports	Flow Stages
Hole detection	26	1	3	11
Surface smoothing	53	4	3	6
Longest-edge subdivision	68	1	3	4
Surface remeshing	81	1	3	38
Mean camber extraction	47	3	4	9
Point-cloud downsampling	30	2	3	8
Stack edge detection	45	2	5	10
Morphological closing	29	1	4	4
Implicit surface extraction	101	8	6	8
Curve-network construction	31	1	6	3

**Table 9:** Serial and deterministic-parallel execution time over ten fresh-process measurements. Times are the mean ± sample standard deviation; speedup is the ratio of execution-time means.

Workflow	Serial (s)	Parallel (s)	Speedup
Point-cloud voxel	4.111 ± 0.011	0.658 ± 0.008	6.25×
Image-stack MIP	46.953 ± 1.538	6.817 ± 0.069	6.89×
Adaptive segmentation	20.207 ± 0.248	9.391 ± 0.115	2.15×

**Table 10:** End-to-end tgVM workflow measurements (N=10). Execution time is the mean ± sample standard deviation; CI is the two-sided 95% Student-*t* interval; RSS is mean peak resident memory.

Workflow	Execution (s)	95% CI (s)	RSS (MiB)
Hole detection	0.194 ± 0.005	[0.190, 0.198]	73.6
Surface smoothing	0.602 ± 0.010	[0.594, 0.609]	203.9
Surface remeshing	0.944 ± 0.010	[0.937, 0.951]	262.9
Mean camber extraction	0.951 ± 0.011	[0.943, 0.959]	302.8
Point-cloud downsampling	4.116 ± 0.010	[4.109, 4.124]	275.8
Stack edge detection	18.941 ± 0.305	[18.723, 19.159]	3758.6
Morphological closing	0.028 ± 0.001	[0.027, 0.028]	41.1
Implicit surface extraction	39.786 ± 0.896	[39.145, 40.427]	7187.0
Curve-network construction	0.001 ± 0.000	[0.001, 0.001]	19.1

**Table 11:** Tracing-collector cost for allocation-heavy parallel workflows (N=3). Values are the mean ± sample standard deviation.

Workflow	Collector	Execution (s)	Peak RSS (MiB)
Point-cloud map/reduce	disabled	0.659 ± 0.019	1323.3 ± 0.6
Point-cloud map/reduce	enabled	2.488 ± 0.021	1496.7 ± 29.6
Image-stack MIP	disabled	6.990 ± 0.022	7699.0 ± 51.5
Image-stack MIP	enabled	13.236 ± 0.204	7940.3 ± 13.0

**Table 12:** Deterministic-output check over ten executions at each worker count.

Workflow	Workers	Within-Platform Result
Point-cloud map/reduce	1, 2, 4, 10	identical
Image-stack map	1, 2, 4, 10	identical
Adaptive-image map	1, 2, 4, 10	identical

## Data Availability

The complete paper workflow sources, released ANTLR4 language grammar, browser-based execution route, downloadable Apptainer/Singularity reproduction image, and instructions for running all reported examples are available at https://tglang.org/reproduce-paper (accessed on 24 August 2026). The datasets used for the hole-detection results are available through Mendeley Data [21]. Software availability and licensing are detailed in Appendix A; the grammar is reproduced in Appendix B.

## Appendix A. Software Availability and Reproduction

Two access routes are provided. First, the programs can be opened and executed through the publicly hosted *tgLang Studio* at https://tglang.org (accessed on 24 August 2026); this route requires only a modern desktop web browser and a network connection, and it does not require a local compiler or container runtime. Second, https://tglang.org/reproduce-paper (accessed on 24 August 2026) provides the complete paper workflow sources, input data that can be redistributed, execution instructions, and a downloadable Apptainer/Singularity image for offline reproduction. The container is the archival route for reproducing the paper with the revised x86-64 Linux build of *tgLang* 1.4.1. The reported timings were collected with the arm64 macOS 1.4.0 build. Version 1.4.1 additionally includes the runtime-teardown correction described in the garbage-collection discussion; this correction occurs after program execution and does not alter the measured algorithms. Table A1 summarizes both access routes and the associated terms.

The reproduction page documents commands for running one workflow, executing the complete batch, controlling worker-thread counts, retaining generated .tgvis, .stl, and point-cloud outputs, and starting the native server mode. Consequently, installing a container runtime is optional for readers who only wish to inspect, modify, and execute the examples; it is needed only for the self-contained offline reproduction route.

**Table A1:** Availability and execution routes for the software artifact.

Item	Availability
Hosted execution	*tgLang Studio* is publicly hosted at https://tglang.org. Each paper program has a dedicated launch link at https://tglang.org/reproduce-paper (accessed on 24 August 2026); opening a link loads the exact source and required input data in Managed Mode. No account or local *tgLang* installation is required. Managed Mode uses the queue-limited native execution service while hiding unrelated authentication and runtime-selection controls. To limit abuse, known VPN and Tor exit nodes cannot use hosted Managed Mode; the offline artifact remains available without this restriction.
Paper source programs	The complete .tg workflow sources used by the paper are available from https://tglang.org/reproduce-paper (accessed on 24 August 2026) and are also bundled in the reproduction image.
Language grammar	The complete ANTLR4 grammar for the evaluated language release is reproduced in Appendix B and distributed as tgLang.g4 with the journal and reproduction artifacts.
Offline artifact	The same page provides tglang-release.sif, an Apptainer/Singularity image containing *tgLang* 1.4.1, the paper programs, redistributable inputs, benchmark evidence, and batch-execution helpers.
Supported platform	The supplied container targets 64-bit x86 Linux and requires Apptainer or Singularity. Native development and validation cover x86-64 Linux and arm64 macOS; no standalone proprietary compiler download is offered for those platforms as part of this artifact. The hosted route is platform-independent at the client side and requires a modern desktop browser. No GPU is required by either reproduction route.
Resource requirements	The complete workflow batch was validated on a 16 GiB machine. Individual programs can be run separately when less memory or execution time is available. Offline reproduction additionally requires space for the 43.6 MB image and generated workflow outputs.
Implementation source	The workflow programs and complete language grammar are source-available as research artifacts. The *tgLang* compiler and runtime implementation source is proprietary and is not publicly distributed; the container supplies the revised x86-64 Linux 1.4.1 build, while the reported timings were obtained with the arm64 macOS 1.4.0 build.
Benchmark evidence	Raw *N* = 10 workflow observations, summary statistics, memory measurements, deterministic-output checks, metric scripts, and the execution-environment manifest are bundled under the paper examples.
Software terms	The *tgLang* compiler and runtime are proprietary research software. The accompanying research-artifact terms permit execution of the unmodified binary/container and inspection or modification of workflow, benchmark, and grammar sources for non-commercial academic evaluation, peer review, teaching, and paper reproduction. All other rights are reserved; this limited permission is not an open-source license.
Build availability	Because implementation source is not released, no compiler build procedure is claimed. The offline artifact is already built and is executed directly with Apptainer/Singularity; commands for individual workflows, the full batch, and benchmark regeneration are supplied.

## Appendix B. tgLang Grammar

Listing A1 gives the complete ANTLR4 grammar used to generate the parser for the evaluated *tgLang* release. It specifies the lexical and context-free syntax accepted by the compiler in the experiments reported here.

The grammar determines syntactic well-formedness only. Name resolution, type and shape validation, overload selection, internal flow-value inference, and bytecode generation are subsequent compiler phases; representation invariants are additionally checked by tgVM and the native runtime. These mechanisms are described in Section 2.1, Section 2.2, and Section 3 and are not inferred from the grammar listing.

**Listing A1:** Complete ANTLR4 grammar used by the evaluated *tgLang* release.
